# Temporal enhancer profiling of parallel lineages identifies AHR and GLIS1 as regulators of mesenchymal multipotency

**DOI:** 10.1093/nar/gky1240

**Published:** 2018-12-14

**Authors:** Deborah Gérard, Florian Schmidt, Aurélien Ginolhac, Martine Schmitz, Rashi Halder, Peter Ebert, Marcel H Schulz, Thomas Sauter, Lasse Sinkkonen

**Affiliations:** 1 Life Sciences Research Unit, University of Luxembourg, L-4367 Belvaux, Luxembourg; 2 Excellence Cluster for Multimodal Computing and Interaction, Saarland Informatics Campus, 66123 Saarbrücken, Germany; 3 Computational Biology & Applied Algorithmics, Max Planck Institute for Informatics, Saarland Informatics Campus, 66123 Saarbrücken, Germany; 4 Molecular Disease Mechanisms Group, Life Sciences Research Unit, University of Luxembourg, L-4367 Belvaux, Luxembourg; 5 Luxembourg Centre for Systems Biomedicine, University of Luxembourg, Esch-sur-Alzette, L-4362, Luxembourg

## Abstract

Temporal data on gene expression and context-specific open chromatin states can improve identification of key transcription factors (TFs) and the gene regulatory networks (GRNs) controlling cellular differentiation. However, their integration remains challenging. Here, we delineate a general approach for data-driven and unbiased identification of key TFs and dynamic GRNs, called EPIC-DREM. We generated time-series transcriptomic and epigenomic profiles during differentiation of mouse multipotent bone marrow stromal cell line (ST2) toward adipocytes and osteoblasts. Using our novel approach we constructed time-resolved GRNs for both lineages and identifed the shared TFs involved in both differentiation processes. To take an alternative approach to prioritize the identified shared regulators, we mapped dynamic super-enhancers in both lineages and associated them to target genes with correlated expression profiles. The combination of the two approaches identified aryl hydrocarbon receptor (AHR) and Glis family zinc finger 1 (GLIS1) as mesenchymal key TFs controlled by dynamic cell type-specific super-enhancers that become repressed in both lineages. AHR and GLIS1 control differentiation-induced genes and their overexpression can inhibit the lineage commitment of the multipotent bone marrow-derived ST2 cells.

## INTRODUCTION

Understanding the gene regulatory interactions underlying cell differentiation and identity has become increasingly important, especially in regenerative medicine. Efficient and specific reprogramming of cells toward desired differentiated cell types relies on understanding of the cell type-specific regulators and their targets (1). Similarly, knowledge of the regulatory wiring in the intermediate stages might allow controlled partial dedifferentiation, and thereby endogenous regeneration, also in mammals (2).

Great progress has been made in reconstruction of GRNs for various cell types in recent years. While successful, many of the approaches derive their regulatory interactions from existing literature and databases, which may be limiting as the majority of enhancers harboring transcription factor (TF) binding sites are cell type-specific (3). Thus, the regulatory interactions derived from existing databases and literature might be misleading and are likely to miss important interactions that have not been observed in other cell types. Therefore, context-specific expression data have been used to overcome such biases and allow a data-driven network reconstruction (4). In addition, other approaches taking advantage of time-series data, such as Dynamic Regulatory Events Miner (DREM) (5), have been developed to allow hierarchical identification of the regulatory interactions. However, while time-series epigenomic data has been used in different studies to derive time point-specific GRNs (6,7), systematic approaches that integrate the different types of data in an intuitive and automated way are missing.

The central key genes of biological networks under multi-way regulation by many TFs and signaling pathways were recently shown to be enriched for disease genes and are often controlled through so called super-enhancers (SEs), large regulatory regions characterized by broad signals for enhancer marks like H3 lysine 27 acetylation (H3K27ac) (8–11). Hundreds of SEs can be identified per cell type, many of which are cell type- or lineage-specific and usually control genes that are important for the identity of the given cell type or condition. Thus, SE mapping and SE target identification can facilitate unbiased identification of novel key genes.

An example of lineage specification events with biomedical relevance is the differentiation of multipotent bone marrow stromal progenitor cells toward two mesenchymal cell types: osteoblasts and bone marrow adipocytes. Due to their shared progenitor cells, there is a reciprocal balance in the relationship between osteoblasts and bone marrow adipocytes. Proper osteoblast differentiation and maturation toward osteocytes is important in bone fracture healing and osteoporosis and osteoblast secreted hormones like osteocalcin can influence insulin resistance (12,13). At the same time bone marrow adipocytes, that occupy as much as 70% of the human bone marrow (14), are a major source of hormones promoting metabolic health, including insulin sensitivity (15). Moreover, increased commitment of the progenitors toward the adipogenic lineage upon obesity and aging was recently shown to inhibit both bone healing and the hematopoietic niche (16).

Extensive temporal epigenomic analysis of osteoblastogenesis has been recently reported (17). Moreover, a parallel investigation of adipocytes and osteoblasts differentiated from the same primary bone marrow-derived progenitor cells was performed by Meyer *et al.* (18). Such analysis can help to understand both the lineage-specific and the shared regulators important for their (de)differentiation. To further identify shared regulators of adipocyte and osteoblast commitment, and to delineate a general approach for systematic unbiased identification of key regulators, we performed time-series epigenomic and transcriptomic profiling at six different time points over 15-day differentiation of multipotent bone marrow stromal cell line (ST2 cells) toward both adipocytes and osteoblasts. We combine segmentation-based TF binding predictions from time point-specific active enhancer data (19) with probabilistic modeling of temporal gene expression data (5) to derive dynamic GRNs for both lineages. By merging overlapping SEs identified using H3K27ac signal from different time points we obtained dynamic profiles of SE activity across the two differentiations and use these dynamic SEs to prioritize the key regulators identified through the network reconstruction. With this approach, we identified aryl hydrocarbon receptor (AHR) and Glis family zinc finger 1 (GLIS1) as central regulators of multipotent mesenchymal cells under dynamic control from SEs that become repressed in the differentiated cells. Overexpression of either of the TFs is able to inhibit adipogenesis and GLIS1 can also prevent osteoblast differentiation. The repression of these TFs, and in particular of AHR, allows upregulation of many adipocyte- and osteoblast-specific genes, including *Notch* genes, a family of conserved developmental regulators.

## MATERIALS AND METHODS

### Cell culture

The mouse multipotent bone marrow stromal ST2 cell line, established from Whitlock-Witte type long-term bone marrow culture of BC8 mice (20), was used during all experiments. The hypotetraploid cells were grown in Roswell Park Memorial Institute (RPMI) 1640 medium (Gibco, Life Technologies, 32404014) supplemented with 10% fetal bovine serum (FBS) (Gibco, Life Technologies, 10270–106, lot #41F8430K) and 1% L-Glutamine (Lonza, BE17–605E) in a constant atmosphere of 37°C and 5% CO_2_. All experiments were carried out with cells passaged for less than 10 times. For differentiation into adipocytes and osteoblasts, ST2 cells were seeded 4 days before differentiation (day-4), reached 100% confluency after 48 h (day-2) and were further maintained for 48 h post-confluency (day 0). Adipogenic differentiation was subsequently initiated on day 0 (D0) by adding differentiation medium I consisting of growth medium, 0.5 mM isobutylmethylxanthine (IBMX) (Sigma-Aldrich, I5879), 0.25 μM dexamethasone (DEXA) (Sigma-Aldrich, D4902) and 5 μg/ml insulin (Sigma-Aldrich, I9278). From day 2 (D2) on differentiation medium II consisting of growth medium, 500 nM rosiglitazone (RGZ) (Sigma-Aldrich, R2408) and 5 μg/ml insulin (Sigma-Aldrich, I9278) was added and replaced every 2 days until 15 days of differentiation. Osteoblastic differentiation was induced with growth medium supplemented with 100 ng/ml bone morphogenetic protein-4 (BMP-4) (PeproTech, 315–27). Same media was replaced every 2 days until 15 days of osteoblastogenesis. The differentiation efficiency was controlled by real-time quantitative polymerase chain reaction (RT-qPCR) of established marker genes and by observing cell morphology and Oil Red O or von Kossa stainings (Supplementary Figure S1).

### Generation of stable cell lines

To generate stable ST2 cell lines with integration of *CopGFP* gene, *Ahr* gene or *Glis1* gene under a Tet-On 3G promoter (ST2-TetOn-GFP, ST2-TetOn-AHR, and ST2-TetOn-GLIS1, respectively) for inducible overexpression of the indicated genes, ST2 cell were transduced with lentiviral particles from Sirion Biotech (Germany) at a MOI of 2.0 together with reverse tetracycline transactivator (rtTA) under the control of the mouse cytomegalovirus promoter (mCMV). Transduced ST2 cells were then selected by adding puromycin (InvivoGen, ant-pr-1) to the growth medium at a concentration of 1 μg/ml. Induction of *CopGFP, Ahr* or *Glis1* was achieved by adding doxycycline (Takara, 631311) at a concentration of 1 μg/ml (stock concentration 1 mg/ml in sterile filtered distilled water).

### Gene silencing

Undifferentiated ST2 cells (day-1) were transfected with Lipofectamine RNAiMAX (Life Technologies, 13778150) according to manufacturer's instructions using 50 nM of gene-specific siRNAs against mouse *Ahr* (si*Ahr*) (Dharmacon, M-044066–01-0005), *Glis1* (si*Glis1*) (Dharmacon, M-065576–01-0005) or 50 nM of a negative control siRNA duplexes (si*Control*) (Dharmacon, D-001206–14-05). Cells were collected 48 h post-transfection. Sequences of the siRNAs are listed in Supplementary Table S1.

### Western blotting

After washing the cells with 1× phosphate-buffered saline (PBS), and addition of 1× Läemmli buffer, the lysates were vortexed and the supernatants were heated at 95°C for 7 min. Proteins were subjected to sodium dodecyl sulphate-polyacrylamide gel electrophoresis (SDS-PAGE) (10% gel) and probed with the respective antibodies. The following antibodies were used: anti-AHR (Enzo Life Biosciences, BML-SA210–0025), anti-ACTIN (Merck Millipore, MAB1501). HRP-conjugated secondary antibodies were purchased from Cell Signaling. Signals were detected on a Fusion FX (Vilber Lourmat) imaging platform, using an ECL solution containing 2.5 mM luminol, 100 mM Tris/HCl pH 8.8, 0.2 mM para-coumaric acid, and 2.6 mM hydrogenperoxide.

### RNA extraction and cDNA synthesis

Total RNA was extracted from ST2 cells using TRIsure (Bioline, BIO-38033). Medium was aspirated and 1000 μl of TRIsure was added to 6-wells. To separate RNA from DNA and proteins, 200 μl of chloroform (Carl Roth, 6340.1) was added. To precipitate RNA from the aqueous phase, 400 μl of 100% isopropanol (Carl Roth, 6752.4) was added and RNA was incubated at −20°C overnight. cDNA synthesis was done using 1 μg of total RNA, 0.5 mM dNTPs (ThermoFisher Scientific, R0181), 2.5 μM oligo dT-primer (Eurofins MWG GmbH, Germany), 1 U/μl Ribolock RNase inhibitor (ThermoFisher Scientific, EO0381) and 1 U/μl M-MulV Reverse transcriptase (ThermoFisher Scientific, EP0352) for 1 h at 37°C or 5 U/ μl RevertAid Reverse transcriptase for 1 h at 42°C. The PCR reaction was stopped by incubating samples at 70°C for 10 min.

### Quantitative PCR

RT-qPCR was performed in an Applied Biosystems 7500 Fast Real-Time PCR System and using Thermo Scientific Absolute Blue qPCR SYBR Green Low ROX Mix (ThermoFisher Scientific, AB4322B). In each reaction 5 μl of cDNA, 5 μl of primer pairs (2 μM) and 10 μl of the Absolute Blue qPCR mix were used. The PCR reactions were carried out at the following conditions: 95°C for 15 min followed by 40 cycles of 95°C for 15 s, 55°C for 15 s and 72°C for 30 s. To calculate the gene expression level the 2^−(ΔΔCt)^ method were used where ΔΔCt is equal to (ΔCt_(target gene)_ – ΔCt_(housekeeping gene)_)_tested condition_ - (ΔCt_(target gene)_ – ΔCt_(housekeeping gene)_)_control condition_. *Rpl13a* was used as a stable housekeeping gene and D0, si*Control*, or untreated cells were used as control conditions. Sequences of the primer pairs are listed in Supplementary Table S1.

### Chromatin immunoprecipitation

Chromatin immunoprecipitation of histone modifications was performed on indicated time points of adipocyte and osteoblast differentiation. Cells were grown on 10 cm^2^ dishes. First, chromatin was cross-linked with formaldehyde (Sigma-Aldrich, F8775–25ML) at a final concentration of 1% in the culture media for 8 min at room temperature. Then, the cross-linked reaction was quenched with glycine (Carl Roth, 3908.3) at a final concentration of 125 mM for 5 min at room temperature. The formaldehyde-glycine solution was removed and cells were washed twice with ice-cold PBS (Lonza, BE17–516F) containing cOmplete™ mini Protease Inhibitor (PI) Cocktail (Roche, 11846145001). Then, cells were lysed in 1.7 ml of ice-cold lysis buffer [5 mM 1,4-Piperazinediethanesulfonic acid (PIPES) pH 8.0 (Carl Roth, 9156.3); 85 mM potassium chloride (KCl) (PanReac AppliChem, A2939); 0.5% 4-Nonylphenyl-polyethylene glycol (NP-40) (Fluka Biochemika, 74385)] containing PI and incubated for 30 min on ice. The cell lysates were then centrifuged at 660 × *g* for 10 min at 7°C and the pellet was resuspended in 400 μl of ice-cold shearing buffer [50 mM Tris Base pH 8.1 (Carl Roth, 4855.2); 10 mM ethylenediamine tetraacetic acid (EDTA) (Carl Roth, CN06.3); 0.1% SDS (PanReac Applichem, A7249); 0.5% Sodium deoxycholate (Fluka Biochemika, 30970)] containing PI. Chromatin was sheared with a sonicator (Bioruptor^®^Standard Diagenode, UCD-200TM-EX) during 20 cycles at high intensity (30 s off and 30 s on) for the ST2 cells differentiated into adipocytes and osteoblasts and 25 cycles at high intensity (30 s off and 30 s on) for the ST2 differentiated into osteoblasts for 9 days on. The sheared cell lysate was then centrifuged at 20817 × *g* for 10 min at 7°C and the supernatant containing the sheared chromatin was transferred to a new tube. For each immunoprecipitation 10 μg (for H3K4me3) or 15 μg (for H3K27ac and H3K36me3) of sheared chromatin and 4 μg as input were used. The sheared chromatin was diluted 1:10 with modified RIPA buffer [140 mM NaCl (Carl Roth, 3957.2); 10 mM Tris pH 7.5 (Carl Roth, 4855.2); 1 mM EDTA (Carl Roth, CN06.3); 0.5 mM ethylene glycol-bis(β-amino-ethyl ether)-N,N,N’,N’-tetraacetic acid (EGTA) (Carl Roth, 3054.3); 1% Triton X-100 (Carl Roth, 3051.2); 0.01% SDS (PanReac Applichem, A7249); 0.1% sodium deoxycholate (Fluka Biochemika, 30970)] containing PI. The diluted sheared chromatin was incubated overnight with the recommended amount provided by the manufacturer of an antibody against H3K4me3 (Millipore, 17–614), 5 μg of an antibody against H3K27ac (Abcam, ab4729) or 5 μg of an antibody against H3K36me3 (Abcam, ab9050). The next day, the antibodies were captured using 25 μl of PureProteome™ Protein A Magnetic (PAM) Bead System (Millipore, LSKMAGA10) for 2 h at 4°C on a rotating wheel. After, the PAM beads were captured using a DynaMag™-2 magnetic stand (Life Technologies, 12321D). The supernatant was discarded and the PAM beads were washed twice with 800 μl of Immunoprecipitation wash buffer 1 (IPWB1) [20 mM Tris, pH 8.1 (Carl Roth, 4855.2); 50 mM NaCl (Carl Roth, 3957.2); 2 mM EDTA (Carl Roth, CN06.3); 1% Triton X-100 (Carl Roth, 3051.2); 0.1% SDS (PanReac Applichem, A7249)], once with 800 μl of Immunoprecipitation wash buffer 2 (IPWB2) [10 mM Tris, pH 8.1 (Carl Roth, 4855.2); 150 mM NaCl (Carl Roth, 3957.2); 1 mM EDTA (Carl Roth, CN06.3), 1% NP-40 (Fluka Biochemika, 74385), 1% sodium deoxycholate (Fluka Biochemika, 30970), 250 mM of lithium chloride (LiCl) (Carl Roth, 3739.1)] and twice with 800 μl of Tris-EDTA (TE) buffer [10 mM Tris, pH 8.1 (Carl Roth, 4855.2); 1 mM EDTA (Carl Roth, CN06.3), pH 8.0]. At last, the PAM beads and the inputs were incubated with 100 μl of ChIP elution buffer [0.1 M sodium bicarbonate (NaHCO_3_) (Sigma-Aldrich, S5761); 1% SDS (PanReac Applichem, A7249)]. The cross-linking was reversed by adding 10 μg of RNase A (ThermoFisher, EN0531) and 20 μg of proteinase K (ThermoFisher, EO0491) at 65°C overnight. Then, the eluted chromatin was purified using a MinElute Reaction Cleanup Kit (Qiagen, 28206) according to the manufacturer’s instructions. The DNA concentration was measured using the Qubit^®^ dsDNA HS Assay Kit (ThermoFisher, Q32851) and the Qubit 1.0 fluorometer (Invitrogen, Q32857) according to the manufacturer’s instructions.

The ChIP assays were performed at each time point in three biological replicates, one of which was subjected to high-throughput sequencing. The enrichment profiles at four selected loci were validated on all three biological replicates by ChIP-PCR (data not shown).

### ChIP-Seq

The sequencing of the ChIP samples was done at the Genomics Core Facility in EMBL Heidelberg, Germany. For sequencing, single-end and unstranded reads were used and the samples were processed in an Illumina CBot and sequenced in an Illumina HiSeq 2000 machine. In total, 979 572 918 raw reads were obtained. Raw reads quality was assessed by fastqc [v0.11, (21)]. This quality control unveiled that some reads were containing part of the adapters. Those spurious sequences were cleaned up from the genuine mouse sequences by AdapterRemoval (22) [v1.5]. The PALEOMIX pipeline (23) [v1.0.1] was used for all steps from FASTQ files to BAM files including trimming, mapping, and duplicate marking. This workflow ensures that all files are complete and valid. Retained reads were required to have a minimum length of 25 bp. Bases with unreliable Phred scores (0–2) were trimmed out. In total 31 909 435 reads were discarded (3.26%). Eventually, 947 663 483 reads were retained (96.74%). Trimmed reads were further mapped using BWA (24) [v0.7.10] with the backtrack algorithm dedicated to short sequences. The mouse reference was the mouse genome GRCm38.p3 (mm10, patch 3) downloaded from NCBI. For validating, merging BAM files, and marking duplicates, we used the suite tool Picard [v1.119, (25)]. Duplicates were marked but not removed. Only reads with a mapping quality of 30 were retained to ensure a unique location on the genome resulting in 661 364 143 reads (69.79% of the trimmed reads). The samples with a coverage of less than 8 million reads (mapping quality > 30) were excluded from the downstream analysis. Raw FASTQ and BAM files have been deposited in the European Nucleotide Archive with the accession number PRJEB20933. The ChIP-seq tracks are available at the UCSC Genome Browser (http://genome.ucsc.edu/cgi-bin/hgTracks?hubUrl=https://biostat2.uni.lu/dgerard/hub.txt&genome=mm10).

The ChIP-Seq peaks were called with Model-based analysis of ChIP-Seq (26) (MACS) version 2.1.0 for H3K4me3, with HOMER (27) for H3K27ac, and with SICER (28) version 1.1 for H3K36me3, using input from undifferentiated ST2 cells as control for IPs from D0 cells and input from D5 adipocyte- or osteoblast-differentiated cells for the IPs from the respectively differentiated cells.

### RNA-Seq

**Figure 1.**
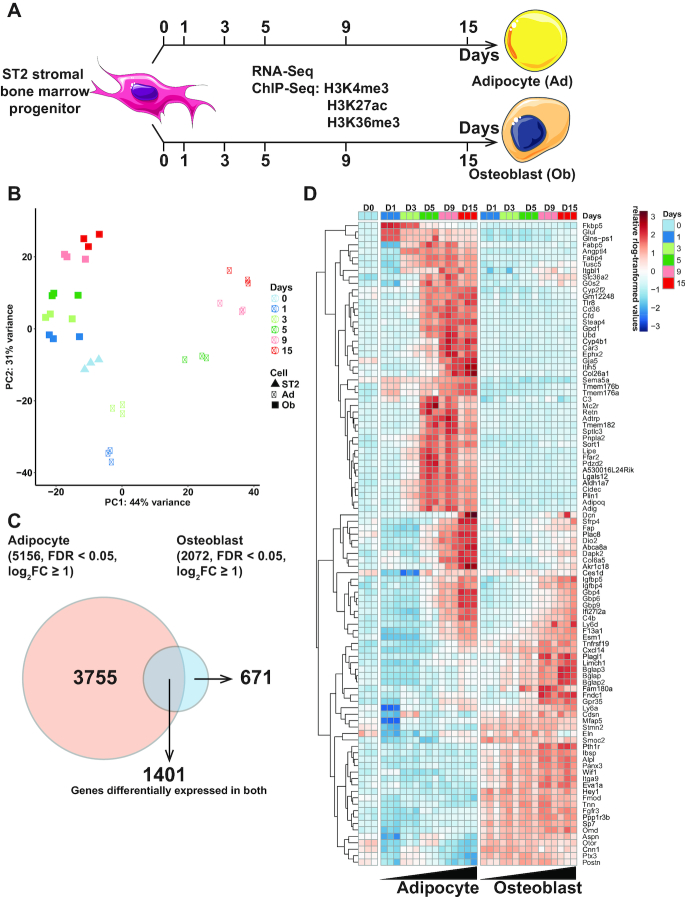
Time-series RNA-seq and ChIP-seq profiling of adipocyte and osteoblast differentiation from shared bone marrow progenitor cells. (**A**) Schematic representation of the experimental set-up. ST2 cells were differentiated toward adipocytes or osteoblasts. Total RNA and chromatin was collected at the indicated time points from both differentiation time courses and subjected to RNA-seq or ChIP-seq analysis using antibodies against the indicated histone modifications. (**B**) Principle component analysis of the RNA-seq data. All replicates are indicated with each time point in a different color. Triangles marks the undifferentiated ST2 cells, circles the adipocyte (Ad) samples and squares the osteoblast (Ob) samples. (**C**) Venn diagram comparing all differentially expressed genes at any time point of adipogenesis (log_2_FC > 1, FDR < 0.05) to those similarly affected in osteoblastogenesis. (**D**) Heatmap depicting the top 100 genes with the highest variance across the time points and lineages.

The sequencing of the time course samples (Figure 1) was done at the Genomics Core Facility in EMBL Heidelberg, Germany. For sequencing, single-end and unstranded reads were used and the samples were processed in an Illumina CBot and sequenced in an Illumina NextSeq machine.

The sequencing of the AHR knock-down samples was performed at the Luxembourg Center for Systems Biomedicine (LCSB) Sequencing Facility. The TruSeq Stranded mRNA Library Prep kit (Illumina) was used to prepare the library for sequencing with 1 μg of RNA as starting material according to manufacturer’s protocol. The library quality was checked using an Agilent 2100 Bioanalyzer and quantified using Qubit dsDNA HS assay Kit. The libraries were then adjusted to 4 nM and sequenced on a NextSeq 500 (Illumina) according to the manufacturer's instructions.

The obtained reads were quality checked using FastQC version 0.11.3 (21). Cutadapt version 1.8.1 (29) was used to trim low quality reads (-q 30 parameter), remove Illumina adapters (-a parameter), remove reads shorter than 20 bases (-m 20 parameter) with an error tolerance of 10% (-e 0.1 parameter). Then, removal of reads mapping to rRNA species was performed using SortMeRNA (30) with the parameters –other, –log, -a, -v, –fastx enabled. Lastly, the reads were quality checked using FastQC version 0.11.3 to control whether bias could have been introduced after the removal of Illumina adapters, low quality reads and rRNA reads. Then, the reads were mapped to the mouse genome mm10 (GRCm38.p3) and using the gene annotation downloaded from Ensembl (release 79) using the Spliced Transcripts Alignment to a Reference (31) (STAR) version 2.5.2b using the previously described parameters (32). The reads were counted using the function *featureCounts* from the R package *Rsubread* (33) version 1.4.6-p3 and the statistical analysis was performed using DESeq2 (34) version 1.14.1 in R 3.3.2 and RStudio (RStudio Team (2015). RStudio: Integrated Development for R. RStudio, Inc., Boston, MA, USA).

### EPIC-DREM analysis

To identify TFs that have a regulatory function over time, we designed a new computational workflow that combines the computational TF prediction method TEPIC (19) with DREM (5), a tool to analyze the dynamics of transcriptional regulation.

We identified TF footprints in the H3K27ac signal using HINT-BC (35), which is included in the Regulatory Genomics Toolbox, version 0.9.9. Next, we predicted TF binding in those footprints using TEPIC, version 2.0 (36).We used the provided set of 687 PWMs for *Mus musculus* and mouse genome version mm10 (GRCm38) to predict TF affinities using TRAP (37) within TEPIC. As DREM requires a time point-specific prediction of binding of a regulator with its target, we needed to develop an approach to determine a suitable TF-specific affinity cut-off, for each time point. For this, we created a similar set of random regions that mirrors the GC content and length distribution of the original sequences of the footprints. TF affinities ${a_r}$ calculated in the random regions are used to determine a suitable cut-off for the original affinities ${a_o}$ using the frequency distribution of the TF affinities. Affinities for $T{F_i}$ are denoted by ${a_{{r_i}}}$ and ${a_{{o_i}}}$. Let $r \in R$ denote a randomly chosen genomic region that is screened for TF binding, and let $| r |$ denote its length. Analogously, let $o \in O$ denote a footprint that is screened for TF binding, and let $| o |$ denote its length. We normalize both ${a_{{r_i}}}$ and ${a_{{o_i}}}$ by the length of their corresponding region and obtain the normalized TF affinities $a^\prime_{r_i}$ and $a^\prime_{o_i}$:

\begin{equation*}\ a^\prime_{r_i} = \ \frac{{{a_{{r_i}}}}}{{\left| r \right|}},\ \ a^\prime_{o_i} = \ \frac{{{a_{{o_i}}}}}{{\left| r \right|}}.\end{equation*}

Using the distribution of $a^\prime_{r_i}$ values we derive a TF-specific affinity threshold ${t_i}$ for a *P*-value cut-off of 0.05 (See section *ChIP-seq validation of TEPIC affinity cut-off* for how this *P*-value was chosen). For a $T{F_i}$, we compute a binary affinity value ${b_{{o_i}}}$ from the original affinity $a^\prime_{o_i}$ according to the cut-off ${t_i}$ with:

\begin{equation*}\ {b_{{o_i}}} = \ \left\{ {\begin{array}{@{}*{1}{c}@{}} {1,\ a^\prime_{o_i} >{t_i}}\\ {0,\ a^\prime_{o_i} \le {t_i}} \end{array}} \right.\end{equation*}

The binary affinity values${b_{oi}}$ can be used to compute a binary TF–gene association ${a_{gi}}$ between gene *g* and TF *i*:


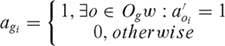
, where ${O_{gw}}$ denotes all footprint regions that occur within a window of size $w$ around the TSS of gene $g$.

Informally, a gene $g$ is associated to $T{F_i}$ if there is a predicted binding site within a window of predefined size ${\rm{w}}$ around the gene’s TSS. Here, we use ${\rm{w\ }} = {\rm{\ }}50{\rm{kb}}$.

**Figure 2.**
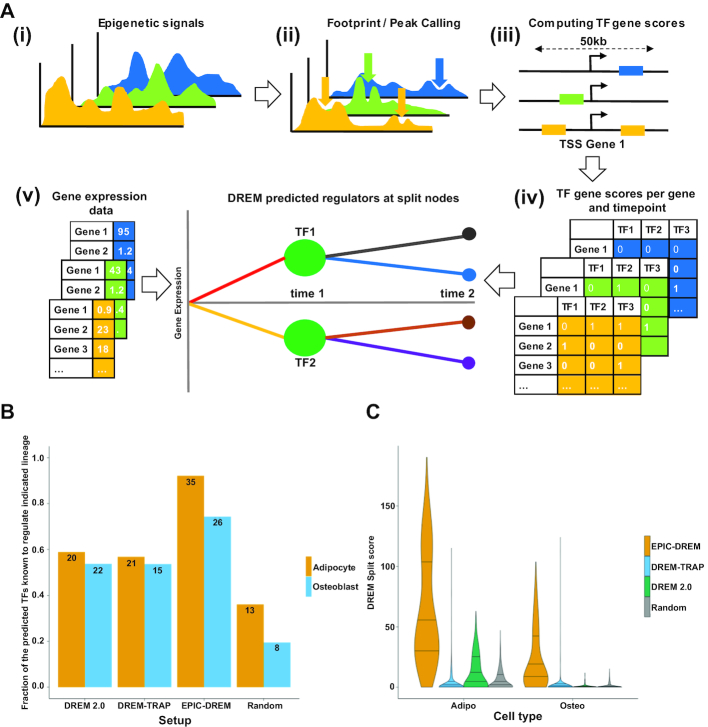
Workflow of the EPIC-DREM approach and benchmarking against other methods. (**A**) (i–iii) From time-series epigenetic experiments, e.g. DNase1-seq or histone ChIP-seq, putative TF binding sites are identified through footprint/peak calling and are annotated with TF affinities using TRAP. Random genomic regions with similar genomic characteristics (GC-content and length) compared to measured footprints/peaks are generated and annotated with TF affinities as well. A TF- and timepoint-specific affinity threshold can be obtained by applying an empirical *P*-value threshold (e.g. 0.05) on the distribution of TF affinities calculated on the randomly selected regions. Applying the threshold on the TF affinities computed in (iii) results in a set of discrete TF affinities per TF and timepoint. (iv) Using TEPIC, the discrete TF affinities are integrated into discrete, timepoint-specific TF–gene association scores. (v) The discrete, time-point specific TF–gene association scores together with the time-series gene-expression data are used within DREM to predict regulators that distinguish subsets of genes showing similar gene expression changes over time. Here, at the beginning of the time course, DREM identifies two sets of genes, denoted by the red and yellow lines, denoting two distinct expression patterns that are associated to TF1 and TF2. Going on to the next time point, DREM suggests that the red and yellow gene sets form two subgroups each, black and blue, as well as brown and purple, respectively. TF binding predictions for EPIC-DREM have been included in the TEPIC 2.0 software (36) (**B**) Benchmarking of the different methods. The time-series data from ST2 cell differentation to adipocytes and osteoblasts (Figure 1A) was used as input for four different approaches (DREM2.0, EPIC-DREM, DREM-TRAP and random TF–target assignment) to identify TFs controlling the respective lineages. Per time point, we consider the top 15 TFs ranked according to their DREM score. For each identified TF the literature was searched for existing evidence for that TFs role in maintaining mesenchymal stem cells or for its involvement in differentiation to either adipocytes or osteoblasts. Supplementary Table S4 provides the retrieved literature references. The fraction of the predicted TFs for which we found supporting literature is shown per method and lineage, with the total number of identified unique top TFs indicated. (**C**) The distributions of the obtained DREM split scores per method and lineage are shown, with EPIC-DREM typically obtaining highest scores at most split points.

Together with gene expression estimates, the TF–gene associations can be directly used as input to DREM. In this analysis, we used version 2.0.3 of DREM. The entire workflow of EPIC-DREM is shown in Figure 2A. TEPIC is available online at https://github.com/SchulzLab/TEPIC, DREM can be downloaded at http://www.sb.cs.cmu.edu/drem/.

### ChIP-seq validation of TEPIC affinity cut-off

To validate that the affinity threshold described above indeed results in an adequate separation between bound and unbound sites, we conducted a comparison to TF-ChIP-seq data. We obtained TF-ChIP-seq data from ENCODE for K562 (18 TFs), HepG2 (36 TFs) and GM12878 (24 TFs). In addition, we downloaded H3K27ac data for the mentioned cell lines from ENCODE. A list of all ENCODE accession numbers is provided in Supplementary Table S2. As described above, we called footprints using HINT-BC and calculated TF affinities in the footprints as well as in the randomly selected regions that map the characteristics of the footprints. To understand the influence of different thresholds, we calculated affinity thresholds for the following *P*-values: 0.01, 0.025, 0.05, 0.075, 0.1, 0.2, 0.3, 0.4 and 0.5. All affinities below the selected affinity value are set to zero, the remaining values are set to one. The quality of the discretization is assessed through the following ‘peak centric’ validation scheme, as used before in (38). The positive set of the gold standard is comprised of all ChIP-seq peaks that contain a motif predicted by FIMO (39), the negative set contains all remaining ChIP-seq peaks. A prediction is counting as a true positive (TP) if it overlaps the positive set, it counts as a false positive (FP) it if overlaps the negative set. The number of false negatives (FN) is the number of all entries in the positive set that are not overlapped by any prediction. For all TFs in all cell lines we calculate Precision (PR) and Recall (REC) according to

\begin{equation*}{\rm PR}\ = \frac{{{\rm TP}}}{{{\rm TP }+ {\rm FP}}}\ ,\ {\rm REC}\ = \frac{{{\rm TP}}}{{{\rm TP }+ {\rm FN}}}\ .\end{equation*}

As one can see, Precision is increasing with a stricter *P*-value threshold, while Recall is decreasing. We found that using 0.05 seems to be a reasonable compromise between Precision and Recall. The median Precision and Recall values calculated over all cell lines and all TFs are shown in Supplementary Figure S2A. Detailed results on a selection of TFs that are present in all three cell lines are shown in Supplementary Figure S2B.

### Generating TF–TF interaction networks for DREM splits

We devised a general strategy to create TF–TF interaction networks for individual DREM splits. For a split of interest, we retrieve the top 25 regulators $T$, ranked according to the DREM split score. For each regulator ${\rm{t}} \in {\rm{T}}$, we determine the target genes ${G_t}$ by declaring each gene *g* to be a target of $T{F_t}$, if and only if ${{\rm{a}}_{{\rm{g}},{\rm{t}}}} = {\rm{\ }}1$, where ${{\rm{a}}_{{\rm{g}},{\rm{t}}}}$ is the binary value for the TF affinity of $T{F_t}$in the vicinity of gene $g$, as introduced above. Here, we want to show only interactions among the top regulators, thus, in order to include a directed edge from $t$ to $g$ in the TF–TF network, it must hold that ${\rm{g}} \in {\rm{T}}$. For reasons of simplicity, we limit the number of shown interactions per $T{F_t}$ to 10, ranked by the numerical affinity values across all ${\rm{\ g}} \in {{\rm{G}}_{\rm{t}}}$.

Additionally, we scale the size of each node representing a regulator ${\rm{t}} \in {\rm{T}}$, according to its total number of target genes $|{G_t}$| |in six discrete levels (<2000, <4000, <6000, <8000, <10 000 and >10 000). This allows an interpretation of the importance of the regulator outside the scope of the interactions among the top TFs for the shown split. The node color indicates the expression change of the depicted TF compared to time-point zero. Orange refers to downregulation, whereas blue refers to upregulation.

The networks are arranged with *graphviz* and the *neato* layout algorithm (40).

### DREM-TRAP and random TF–gene assignment

To assess the impact of epigenetics data on DREM predictions, we designed a baseline approach, assuming that most transcriptional regulation for a gene is occurring in its promoter region. Hence, for each gene *g*, we consider a 2 kb window around the most 5′ TSS of *g*. Within this window, we compute TF affinities as described above for 687 PWMs for *M. musculus* and mouse genome version mm10 (GRCm38). Also, we applied the same thresholding approach as described for EPIC-DREM. Thereby, DREM-TRAP represents approaches that are purely sequence and annotation based and do not account for changes in the chromatin.

As another sanity check for EPIC-DREM, we permuted the EPIC-DREM input matrix, which is based on time-point specific footprint calls. In detail, we randomly shuffled column 1 (TF), column 2 (target gene) and column 4 (time point). Thus the number of TFs, target genes and timepoint entries is not affected, only the mapping between them changed.

### Enrichment score of DREM splits, its aggregation and literature evaluation of suggested regulators

DREM uses a hypergeometric distribution to assess the association of a TF to genes in a distinct path, the so called split-score, where a lower value means a stronger association. Because we carry out a considerable number of tests per split (as we test more than 600 TFs), we correct the split-scores for multiple testing using Bonferroni correction.

To compare the enrichment scores across the different versions of DREM used in this manuscript, we –log2 transformed the Bonferroni corrected split-scores and show violin plots of the transformed scores, which we call *DREM split score*. For those scores, the higher the value, the better is the association of the TFs identified at split points to genes on the adjacent paths.

To identify essential TFs across multiple splits at one time point, we perform *P*-value aggregation using Fisher’s method on the split scores, i.e. we compute

\begin{equation*}\ {X_{t.n}} = \ - 2\mathop \sum \limits_{i\ = \ 1}^s {\rm{log}}\left( {{p_{n,i,t}}} \right)\end{equation*}

Here, ${{\rm{X}}_{{\rm{t}},{\rm{n}}}}$ is the aggregated score for $T{F_t}$ at time point $n$, $i$ is the current split, $s$ indicates the number of splits at Timepoint $n$ and ${{\rm{p}}_{{\rm{n}},{\rm{i}},{\rm{t}}}}$ is the split score for $T{F_t}$ at split $i$ at time point $n$. Next, we rank the, ${{\rm{X}}_{{\rm{t}},{\rm{n}}}}$ by time point $n$ and thereby obtain a list of top regulators per time point.

We obtained such lists for DREM2.0, EPIC-DREM, DREM-TRAP and the random TF–gene assignment delineated in the previous section. For each time point and method, we consider the top 15 TFs and screened literature to learn about whether these TFs are known to play a role in mesenchmymal stem cells or in differentiation of either adipocytes or osteoblasts. Note that in case a TF is selected in multiple time points, it counts only once. To directly compare the quality of the predictions between the DREM versions, we compute the precision of the predictions by dividing the number of TFs with existing supportive literature by the total number of identified top regulators.

### Identification of dynamic merged SEs

In order to identify temporal SEs across both lineages, BedTools (41) version 2.24.0, Hypergeometric Optimization of Motif EnRichment (27) (HOMER) version 4.7.2 and Short Time-series Expression Miner (42) (STEM) version 1.3.8 were used. First, the coverage of individual SEs was summarized using genomeCoverageBed command using –g mm10 and –bg parameters. Then, unionBedGraphs command was used to combine multiple SEs coverage into a single map such that the SEs’ coverage is directly comparable across multiple samples. At last, mergeBed command was used to combine SEs overlapping ≥ 1 bp into a single merged SE which spans all the combined SEs. In order to calculate the normalized read count number of merged SEs, annotatePeaks.pl with –size given and –noann parameters was used. At last, STEM was used to cluster and identify SEs temporal profiles and SEs with *Maximum_Unit_Change_in_Model_Profiles_between_Time_Points* 2 and *Minimum Absolute Expression Change* 1.0 were considered as dynamic.

For the dynamic SEs we calculated the Pearson correlation for the putative target genes with their TSS located within ±500 kb, and associated the SEs to the genes with the highest correlation coefficient.

### Enrichment analysis

EnrichR (14.4.2017) (43,44) was used to perform gene enrichment analysis.

## RESULTS

### A subset of differentially expressed genes are shared between adipocyte and osteoblast differentiation

To identify shared regulators of differentiation toward adipocytes and osteoblasts, and to delineate a general approach for a systematic unbiased identification of key regulators, we performed time-series ChIP-seq and RNA-seq profiling at six different time points over 15 days of differentiation of mouse ST2 cells (Figure 1A). Using ChIP-seq, genome-wide profiles of three different histone modifications, indicating active transcription start sites (TSS) (H3K4me3), active enhancers (H3K27ac) and on-going transcription (H3K36me3) were generated. These data were complemented by corresponding time-series RNA-seq analysis. Importantly, at genome-wide level all the histone modifications showed good correlation with the RNA-seq data across the time points (Pearson correlation co-efficients of ∼0.5), further arguing for the reproducibility of the obtained results (data not shown). The successful differentiations were confirmed by induced expression of known lineage-specific marker genes and microscopic inspection of cellular morphology and stainings (Supplementary Figure S1). Interestingly, profiles of the adipogenic marker genes resembled those reported for the yellow adipose tissue (YAT) found in the bone marrow, rather than classic white adipose tissue (WAT) (45), consistent with ST2 cells originating from bone marrow stroma (Supplementary Figure S1A). Moreover, the expression profiles of *Sp7* and *Runx2* were consistent to those previously reported for mouse osteoblasts (46).

Principal component analysis of the obtained transcriptome profiles confirmed the specification of the cells toward two different lineages with differential temporal dynamics (Figure 1B). Osteoblastogenesis was accompanied by gradual and consistent progression toward a more differentiated cell type while adipogenesis showed more complex dynamics with a big transcriptome shift after one day of differentiation, followed by a more gradual progression during the following days. This is in keeping with the change in the composition of the differentiation medium from day 2 onward (see ‘Materials and Methods’ section for details). In total the adipocyte differentiation was characterized by a total of 5156 significantly differentially expressed genes ($lo{g_2}FC \ge 1,\ FDR < 0.05)$ across the time series compared to the undifferentiated cells (Figure 1C and Supplementary Table S3). During osteoblast differentiation 2072 genes were differentially expressed at least at one time point. A total of 1401 of these genes were affected in both lineages. However, as illustrated by the top 100 genes with highest variance across the time points, which are depicted in the heatmap in Figure 1D, most genes exhibit either lineage-specific or opposing behavior between the lineages. Only a subset of genes showed similar changes in both lineages (Figure 1D). Thus, narrowing the list of genes that could serve as shared regulators of both differentiation or dedifferentiation processes.

### Unbiased data-driven derivation of context-specific dynamic regulatory networks of adipocyte and osteoblast differentiation using EPIC-DREM

In order to take an unbiased and data-driven approach that can benefit from the time series profiles, we have developed a new method to predict condition-specific TF binding using footprint calling in H3K27ac data and TF motif annotation (35) (37). We previously found that using footprints works well for the prediction of gene expression using TEPIC (19). Our approach uses a randomization strategy, which accurately accounts for differences in footprint lengths and GC-content bias, to assess the significance of TF binding affinity values for each condition or time point (Figure 2A; see ‘Materials and Methods’ section for more details). These time point-specific predictions can be combined with the DREM approach (5) to construct lineage-specific networks that are supported by epigenetics data (called EPIC-DREM, Figure 2A).

At first we have used 78 TF ChIP-seq datasets from three ENCODE cell lines (GM12878, HepG2 and K562) to test the ability of our approach to prioritize condition-specific TF binding sites using this pipeline. Using a *P*-value cutoff < 0.05 we obtained accurate cell-type specific TF binding predictions with a median precision of ∼70% without a major decrease in recall (Supplementary Figure S2) and, thus, used the same cut-off for our time-series differentiation data.

We used EPIC-DREM to analyze all timepoints of the two differentiation time series of adipogenesis and osteoblastogenesis. Depending on the time point and lineage we predicted 0.6 to 1.4 million footprints per time point, consistent with previous reports on the presence of ∼1.1 million DNase-seq footprints per cell type (47). These footprints were annotated with TFs that are expressed during the differentiation and were associated to target genes within 50 kb of their most 5′TSSs to obtain the TF scores per gene and per time point (see ‘Materials and Methods’ section and Figure 2). The full matrix of the time point-specific TF–target gene interactions per lineage can be downloaded at doi:10.5061/dryad.r32t3.

**Figure 3.**
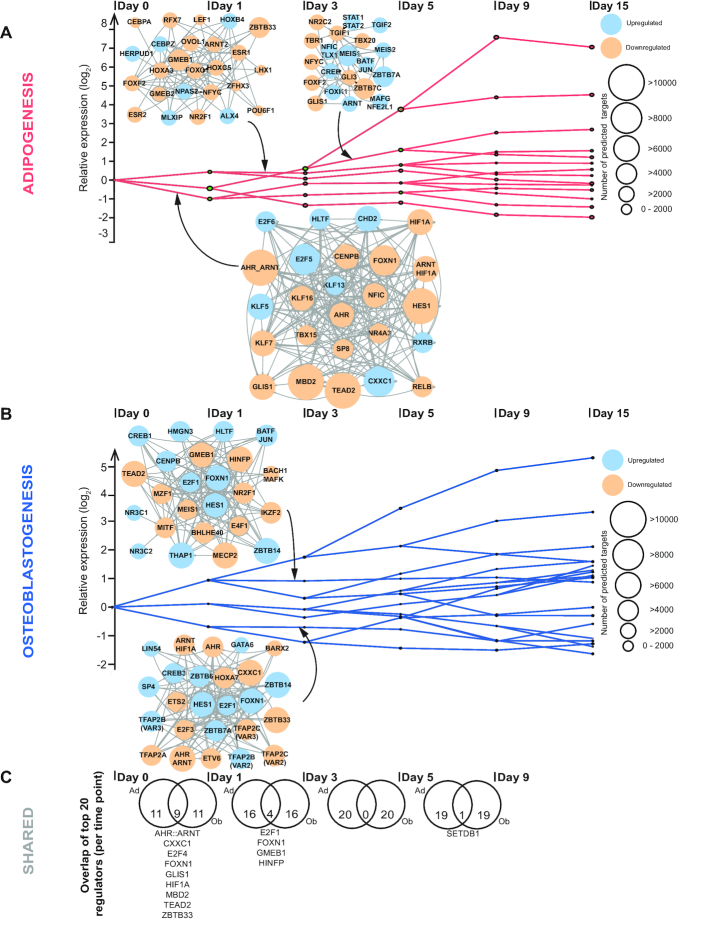
Derivation of lineage-specific regulatory interactions using EPIC-DREM. EPIC-DREM clusters genes into paths of similar expression over time in (**A**) adipocyte and (**B**) osteoblast differentiation. Split points differentiate time point-specific changes in gene expression, that are annotated by DREM with matching TFs based on the predicted TF–target gene interactions. For each such path a GRN of thousands of interactions has been derived. The top TFs based on their time point- and path-specific split scores can be used to generate TF–TF networks underlying the observed gene regulation. The TF–TF networks of top 25 TFs are indicated for (A) three selected paths in adipocyte and (B) two selected paths in osteoblast differentiation. The top TFs are colored depending whether they are upregulated (blue) or downregulated (orange) compared to undifferentiated cells and the size of the node corresponds to the total number of predicted target at the indicated time point. The full set of TFs predicted to control each path with at least 30% of the genes in the upstream split point with a split score ≤ 0.01 are listed in Supplementary Table S5. (**C**) Overlap of the time point-specific top TFs between the lineages. For both lineages lists of top 20 TFs per time point were generated by combining the predictions from individual splits. The Venn diagrams indicate the extent of overlap between the lineage-specific top TFs at each time point and the shared top TFs are indicated.

The derived matrix of the predicted time point-specific TF–target gene interactions was combined with the time series gene expression data to serve as input for DREM to identify bifurcation events, where genes split into paths of co-expressed genes (Figures 2 and 3). Knowing the time point-specific TF–target gene interactions allows an accurate association of split points and paths with the key TFs regulating them. To directly test the accuracy and the biological relevance of the EPIC-DREM predictions compared to alternative methods, we performed the same analysis of the time-series expression data also with three alternative approaches for the prediction of TF–gene interactions: (i) based on DREM 2.0, using existing, time-point unspecific ChIP-seq datasets of TF binding sites; (ii) DREM-TRAP, where the TF binding site predictions are computed in 2 kb windows centered at the gene TSSs without the time- and context-specific epigenomic profiles, and; (iii) Random shuffling of the EPIC-DREM TF–gene interaction matrix (Figure 2B and C). To address the biological relevance of these predictions, we considered the top 15 TFs with best split scores from each split at day 0 and combined them into lists of potential master regulators identified for both lineages and each prediction method (Figure 2B and Supplementary Table S4). Next we performed a literature search for each of the predicted TFs on these lists to see whether they have already been associated with adipogenesis or osteoblastogenesis (Supplementary Table S4). From the randomly assigned TFs only 20–30% had been previously found to be associated with the two cell types, while for the TFs predicted by the time-point unspecific methods DREM 2.0 or DREM-TRAP this fraction increased to between 53% and 59%, depending on the lineage (Figure 2B). However, applying EPIC-DREM for the prediction further improved the fraction of biologically relevant known TFs to as high as 92% for adipocyte and 74% for osteoblast differentiation, respectively. Consistently, the DREM enrichment scores obtained by EPIC-DREM were overall higher than those obtained by the other methods (Figure 2C), indicating that the TFs identified by EPIC-DREM can much better explain the observed expression dynamics.

DREM clusters co-expressed genes along the time-points and identifies split points with transcriptional regulators assigned to them. Figure 3 shows the split points and the paths of co-expressed genes identified for adipocyte (Figure 3A) and osteoblast (Figure 3B) differentiation. The entire predicted gene regulatory networks consist of up to tens of thousands of nodes and edges, preventing their illustration in an intuitive fashion. However, for the selected DREM paths the TF–TF networks of the top 25 TFs (based on their split score) are illustrated in Figure 3, with the size of the TF nodes corresponding to the total number of time point-specific predicted target genes across the genome. The full list of TFs controlling the individual paths are provided in Supplementary Table S5 and the full matrix of the gene regulatory networks can be downloaded at doi:10.5061/dryad.r32t3.

Inspection of the TF–TF networks of the identified top TFs confirmed many known positive (e.g. KLF5 on day 0, CEBPA on D1 and TGIF2 on D3) and negative (e.g. HES1 and NR4A3 on day 0, FOXC1 on D1) regulators of adipocyte differentiation (48–51) (Figure 3A), consistently with the above-mentioned findings that most of the top TFs have been associated with adipogenesis in the literature (Supplementary Table S4). Focusing on the size of the individual nodes in the TF–TF networks directly reveals that TFs controlling the first time point directly after differentiation initiation have the highest number of predicted target genes, with some TFs such as HES1 having more that 10 000 predicted target genes (Figure 3A). Similarly, the results of the regulatory network of osteoblastogenesis confirms several known positive regulators such as the aforementioned HES1, TEAD2 and BHLHE40 (52–54), while revealing many other factors that have not been previously associated to osteoblast differentiation (Figure 3B).

Curiously, EPIC-DREM analysis did not highlight TFs such as PPARG among the top 25 TFs at any bifurcation point, despite PPARG being a well-established master regulator of adipocyte differentiation (55). To better understand this result and to further explore capabilities of EPIC-DREM we had a detailed look at the predictions related to PPARG and the PPARG:RXRA heterodimer. Indeed, both the PPARG monomer and the heterodimer with RXRA appear among the predicted regulators at most split points (see Supplementary Table S5), in particular for the paths of genes upregulated in adipogenesis. The highest ranking of PPARG is obtained for the genes induced directly after differentiation initiation on day 0, where it is ranked as TF number 34 based on the split score. To better illustrate PPARG regulation in yellow adipocyte differentation, we generated a PPARG-centric GRN on day 3 of differentiation (Supplementary Figure S3), the time point where the highest induction of *Pparg* mRNA takes place (Supplementary Figure S1). Supplementary Figure S3 shows all the TFs predicted to control *Pparg* expression at this time point and the top 200 targets out of the total of 3405 genes predicted to be regulated by PPARG:RXRA (Supplementary Table S6). To see whether these predicted targets are consistent with known direct target genes, we used results from Lefterova *et al.* who experimentally identified PPARG targets as genes with at least 3-fold change in their expression in differentiated adipocytes and with binding of PPARG within 50 kb from the TSS based on ChIP-seq data (56). Importantly, our predicted PPARG:RXRA targets were significantly enriched for these experimentally validated targets (hypergeometric test *P* = 2.78e-31).

The fact that PPARG is missing from the lists of the very top TFs explaining the gene expression dynamics during adipogenesis could be due to its modest induction during ST2 cells differentiation (Supplementary Figure S1). Indeed, *Pparg* was noted already in earlier work to be weaker induced in bone marrow derived ST2 cells than in the 3T3-L1 cells that are more commonly used to study white adipocyte differentiation (57). In keeping with these observations, analysis of the histone modifications associated with active transcription at the *Pparg* locus in published data from 3T3-L1 cells confirmed the appearance of strong enhancers (based on H3K27ac signal), increased transcription (H3K36me3) and activation of both alternative transcription start sites (H3K4me3) after differentiation initiation (Supplementary Figure S4; (58)). In contrast, in ST2 cells *Pparg* transcription was abundant already prior to differentiation (H3K36me3), with only one TSS marked by H3K4me3 (transcript variant 1) and strong enhancer regions active across the locus (H3K27ac) (Supplementary Figure S4A). Moreover, while also SE regions could be identified at the *Pparg* locus, consistently with previous work (9), these did not change significantly during differentiation. Thus, our data confirms PPARG as an important SE-controlled regulator with thousands of putative targets in bone marrow-derived ST2 cells and adipocytes differentiated from them, albeit with only modest changes during differentiation.

To see whether the more modest *Pparg* induction is unique to the ST2 cell line or whether it is a general feature of multipotent bone marrow progenitors, we inspected recently published RNA-seq and ChIP-seq from primary mouse bone marrow-derived mesenchymal stem cells and from adipocytes and osteoblasts differentiated from them (18). Also in the primary bone marrow-derived cells *Pparg* induction remained lower than in 3T3-L1 cells with 3- to 4-fold increase after 7 days of adipogenesis, with only TSS of transcript variant 1 marked by H3K4me3 and enhancer signals marked by H3K9ac and H3K4me1 increasing at regions corresponding to *Pparg* SE in ST2 cells (Supplementary Figure S4, see ‘Materials and Methods’ section for UCSC Genome Browser link for the uploaded data from (18)). Thus, *Pparg* regulation in primary bone-marrow derived cells resembles that in ST2 cells, albeit with stronger induction in histone modifications indicating transcriptional activity upon differentiation.

One of our main aims in analyzing two parallel lineages was the identification of shared regulators that could control the differentiation or dedifferentiation of both cell types. With this aim in mind, we combined the predictions from all bifurcation points per time point to generate separate lists of top 20 TFs with most targets for each time point in both lineages (Figure 3C, see ‘Materials and Methods’ section). Next the top 20 TFs at corresponding time points in both lineages were overlapped to uncover the extent and identity of the shared top TFs. As might be expected, the highest level of overlap between the regulators was observed at early differentiation with 9 out of 20 top TFs being same for both lineages (Figure 3C). These are likely to include TFs important for maintaining the multipotent state of the cells and thus, negative regulators of both differentiation processes. Indeed, TFs like the AHR:ARNT heterodimer, E2F4, GLIS1, HIF1A, and TEAD2 have already been associated with gene regulation in different stem cell types (59–65). Interestingly, at day 1 of differentiation the number of shared TFs decreases to four and at later time points the only shared factor is SETDB1, better known as a co-regulator (66). The only TF to appear as shared regulator at two different time points is FOXN1, which is highly connected in GRNs of both lineages, strongly downregulated in adipogenesis but highly expressed in osteoblasts, suggesting FOXN1 might play opposite roles in the two lineages (Figure 3 and Supplementary Table S3).

Taken together, the EPIC-DREM approach can identify many known key regulators of adipocyte and osteoblast differentiation and predicts additional novel regulators and the bifurcation events they control in an entirely unbiased manner relying only on the available time series data.

### Identification of dynamic SEs in adipocyte and osteoblast differentiation

**Figure 4.**
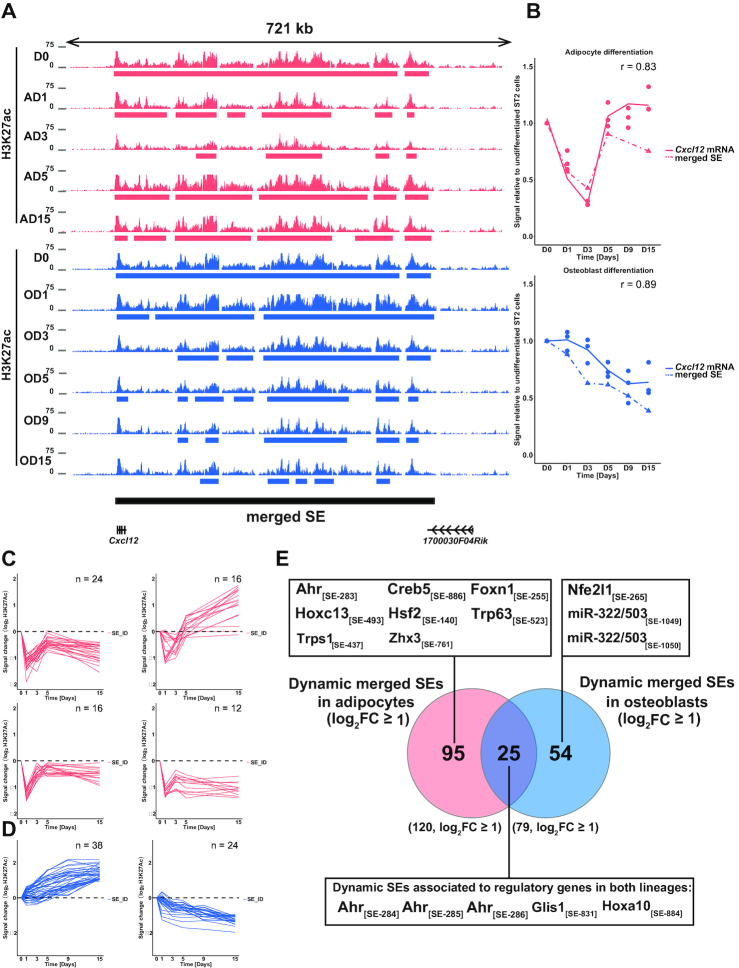
Identification of dynamic SEs. (**A**) Overview depicting the enrichment of H3K27ac at the *Cxcl12* locus across the time points of adipogenesis (in magenta) and osteoblastogenesis (in light blue). The magenta and light blue bars indicate the SE regions identified by HOMER in adipogenesis and osteoblastogenesis, respectively, and black bar indicates the merged SE derived through overlapping of the individual SEs across the lineages. (**B**) The correlation between mRNA levels and SE signal. The *Cxcl12* mRNA levels as measured by RNA-seq and the merged SE signal at the *Cxcl12* locus as measured by the reads detected in the H3K27ac IPs are depicted across the time-series of adipocyte (upper panel) and osteoblast (lower panel) differentiation. Intact line = mRNA level and dashed line = merged SE signal. *r* = Pearson correlation co-efficient (**C** and **D**) STEM clustering of the merged SEs according to their dynamic profiles identifies (C) four main profiles in adipogenesis and (D) two main profiles in osteoblastogenesis. The additional profiles are shown in Supplementary Figure S5. *y*-axis indicates the signal change in log_2_-scale. (**E**) Venn analysis of the dynamic merged SEs from both lineages identifies 95 adipocyte-specific, 54 osteoblast-specific and 25 shared dynamic SEs with log_2_FC ≥ 1. The dynamic SEs were associated to their target genes based on Pearson correlation with gene expression levels across the time series and the regulatory genes (TFs and miRNAs) associated with dynamic SEs are indicated in the corresponding boxes. All dynamic SEs and their putative target genes are listed in Supplementary Table S8. Adipocyte D9 sample for H3K27ac was not included in the above analysis due to low number of mappable high quality reads.

EPIC-DREM can efficiently identify the main regulators of the differentiation time courses that control the highest numbers of target genes at each time point and that can best explain the observed expression dynamics. However, prioritizing the identified TFs also through an alternative approach that relies on different criteria is often desirable. In order to take a parallel approach to further narrow down the identified main regulators, we hypothesized that the key TFs of the differentiation processes would be under high regulatory load and controlled by SEs with dynamic profiles. To obtain such profiles of SEs across time points, we first identified all SEs with a width of at least 10 kb separately in each of the 10 H3K27ac ChIP-seq datasets from the two time courses. Next, to allow a quantification of the SE signals across time, while also accounting for the changes in the width of the SEs, we combined all SEs from all time points with at least 1 nt overlap into one broader genomic region, called a merged SE. Figure 4A illustrates how the 49 SEs found at the *Cxcl12* locus, producing a chemokine essential for maintenance of the hematopoietic stem cells (HSCs) (16), can be combined into one exceptionally large merged SE region that enables the quantification of the SE signal across time. The normalized read counts in the merged SE region were collected and normalized relative to D0 (Figure 4B). To confirm that the obtained profile is a reasonable estimate of the SE activity and to see whether it could indeed be regulating the *Cxcl12* gene expression, we compared the SE signal profiles to the *Cxcl12* mRNA profiles. As shown in Figure 4B, in both lineages the SE signal closely followed the mRNA expression profile with Pearson correlation coefficients of 0.83 and 0.89, respectively. Moreover, similar correlations could be seen for most other identified merged SEs, further confirming the applicability of our approach.

In total, we identified 1052 merged SEs across the two lineages (Supplementary Table S7). A total of 120 and 79 merged SEs showed a dynamic profile (log_2_FC ≥ 1 in at least one time point) in adipocyte and osteoblast differentiation, respectively. Of these, 25 dynamic SEs showed concordant changes in both differentiation processes (Figure 4C–E and Supplementary Table S8). Consistent with the different dynamics in transcriptomic changes (Figure 1), the adipocyte SEs could be divided into four separate main profiles based on their dynamics (Figure 4C and Supplementary Figure S5) while most osteoblast SEs could be assigned into one of two simple profiles that either increase or decrease in signal over time (Figure 4D).

In order to identify the genes regulated by these dynamic SEs we calculated the Pearson correlation for all of the dynamic SEs and all of their putative target genes with their TSS located within ±500 kb, and associated the SEs to the genes with the highest correlation coefficient (Supplementary Table S8). Out of the 151 genes we found to be associated with the dynamic SEs within the defined distance, 12 were known regulatory genes, classified either as TFs (67) or microRNA genes (Figure 4E). From the 12 regulatory genes, 7 were associated with SEs dynamic only in adipogenesis, namely, *Creb5, Hoxc13, Hsf2, Trp63, Trps1, Zhx3* and *Foxn1*. From these FOXN1 was already highlighted among the top TFs by EPIC-DREM analysis, while HOXC13 and HSF2 were also predicted to regulate genes induced during adipogenesis (Supplementary Table S5). The regulatory genes associated with SEs dynamic only in osteoblastogenesis were *Nfe2l1* and *miR-322/503*. From these NFE2L1 was induced and, together with its dimerization partner MAFG, predicted by EPIC-DREM as positive regulator of both osteoblast and adipocyte differentiation (Figure 3 and Supplementary Table S5), while miR-322 and miR-503 have been shown to enhance osteogenesis (68,69).

Finally, three of the regulatory genes, *aryl hydrocarbon receptor* (*Ahr*), *GLIS family zinc finger 1* (*Glis1*), and *Homeobox A10* (*Hoxa10*), were associated with SEs dynamic in both lineages (Figure 4E). Interestingly, all three TFs are predicted as regulators of differentiation by EPIC-DREM (Supplementary Table S5), and both AHR and GLIS1 belong to the top TFs found to be shared between the two lineages (Figure 3C). AHR, the AHR:ARNT heterodimer and GLIS1 belong to the TF–TF network controlling genes in early adipogenesis and depicted in Figure 3A. In addition GLIS1 is also included in the TF–TF network controlling genes induced on day 3 of adipogenesis. Similarly, AHR and AHR:ARNT are found in the TF–TF network controlling genes after day 1 of osteoblastogenesis (Figure 3B). Moreover, *Ahr* is associated with four separate merged SEs, more than any other TF in our analysis, and predicted to function upstream of GLIS1 in the undifferentiated cells (Figures 3A and 4E). Therefore, we next focused on *Ahr* and *Glis1* regulation.

### *Ahr* and *Glis1* are controlled by SEs in undifferentiated ST2 cells and repressed with lineage-specific dynamics

**Figure 5.**
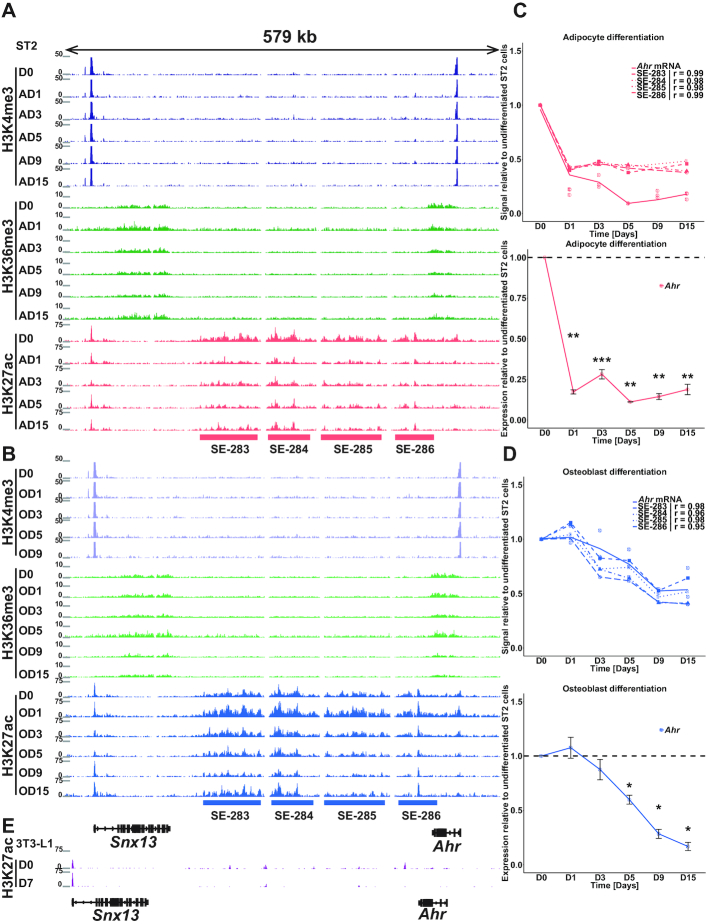
*Ahr* is regulated by multiple SEs with lineage-specific dynamics. (**A** and **B**) Overview depicting the enrichments of H3K4me3 (in dark blue and purple), H3K36me3 (in light and dark green) and H3K27ac (magenta and light blue) at the *Ahr* locus across the time points of adipogenesis (A) and osteoblastogenesis (B), respectively. The magenta and light blue bars indicate the merged SE regions identified through the analysis described in Figure 4. See also Supplementary Figure S7. (**C** and **D**) *Ahr* downregulation correlates with a decreased signal from all four SEs. The *Ahr* mRNA level was measured across the differentiation by RNA-seq (upper panel) and RT-qPCR (lower panel) in both adipocyte (C) and osteoblast (D) differentiation and is indicated as the intact line. Dashed lines represent the signal from the individual indicated merged SEs. *r* = Pearson correlation co-efficient. The statistical significance for RT-qPCR measurements compared to the value on D0 was determined by two-tailed Student’s *t*-test. * = *P*< 0.05, ** = *P*< 0.01 and *** = *P*< 0.001. Data points represent mean of three biological replicates ±SEM. AD9 sample for H3K27ac and OD15 for H3K4me3 were not included in the above analysis due to lower number of mappable high quality reads. (**E**) Overview depicting the enrichment of H3K27ac at the *Ahr* locus in the confluent undifferentiated (D0) and differentiated (D7) 3T3-L1 adipocyte cell line. No SE formation could be detected in these more lineage-committed cells. The data were obtained from (58). The H3K27ac enrichments at the corresponding locus in human cell types are indicated in Supplementary Figure S8A.

The four adjacent merged SEs (SE_283_, SE_284_, SE_285_, and SE_286_) at the *Ahr* locus together cover a continuous region over 300 kb of active enhancer signal downstream of the *Ahr* gene in the ST2 cells (Figure 5A and B). SE_831_ covers a region of 29 kb in the 3′end of the *Glis1* gene in the same cells (Supplementary Figures S6A and B). All four SE regions at the *Ahr* locus follow similar dynamics across the time points (Figure 5A–D) although SE_283_ was identified as being dynamic only in adipogenesis due the FC cut-off in the initial analysis in Figure 4. Moreover, all four SEs showed a very high correlation (*r* ≥ 0.95) with *Ahr* mRNA levels as measured by RNA-seq (Figure 5C and D, upper panels) and validated by RT-qPCR (Figure 5C and D, lower panels). In adipogenesis *Ahr* expression is repressed already by D1 and remains repressed throughout the differentiation, while the signal from all the SE regions is also becoming reduced already after 1 day. In contrast in osteoblasts, the SE regions first show an increase in signal on D1, followed by gradual reduction from D3 onward, consistent with the *Ahr* mRNA levels.

Similarly, the *Glis1* expression changes show high correlation (*r* ≥ 0.95) with the signal of SE_831_ in both lineages and with both RNA-seq and RT-qPCR (Supplementary Figure S6C and D). While in osteoblast differentiation *Glis1* is gradually decreased, in adipocyte differentiation a more dynamic pattern of downregulation is observed with a temporary induction on day 5.

At last, for both genes and in both lineages the repression is accompanied by a decreased signal of H3K36me3 in the gene bodies of the two genes, confirming their repression at the transcriptional level (Figure 5A and B; Supplementary Figure S6A and B). RNA-seq and H3K36me3 ChIP-seq from primary mouse bone marrow-derived mesenchymal stem cells and from adipocytes and osteoblast differentiated from them further confirmed the downregulation of *Glis1* in both lineages and of *Ahr* in adipogenesis (see ‘Data Availability’ section for UCSC Genome Browser link for the uploaded data from Meyer *et al.* (18)). In osteoblastogenesis the decrease of *Ahr* was not confirmed, possibly due to differences in the used differentiation protocols.

While the newly identified SE clusters at the *Ahr* locus are also flanked by another active gene, *Snx13*, it is unlikely that the SEs contribute to its regulation; during the two differentiations *Snx13* shows very few changes in its expression or H3K36me3 signal (Figure 5A and B and data not shown). Moreover, inspection of Hi-C data surrounding the *Ahr* locus in different mouse cell types indicates that *Ahr* and the four SEs are located in their own topological domain (TAD) separate from the *Snx13* gene (Supplementary Figure S7) (70). Similarly, SE_831_ could be confirmed to be located in the same TAD with *Glis1* and the neighboring *Dmrtb1* gene, which is silenced in the ST2 cells, suggesting that *Glis1* is the main target of the SE.

Interestingly, the SEs controlling *Ahr* and *Glis1* appear to be specific for the multipotent cells as only weak or no H3K27ac signal could be detected in the corresponding genomic region in mouse 3T3-L1 pre-adipocytes that are more committed toward the white adipocyte lineage (Figure 5E and Supplementary Figure S6E, (58)). Moreover, the SE regions at both genes were enriched for additional enhancers marks, H3K4me1 and H3K9ac, in primary mouse bone marrow-derived mesenchymal stem cells (see ‘Data Availability’ section for UCSC Genome Browser link for the uploaded data from Meyer *et al.* (18)). Importantly, the large SE domains downstream of the *Ahr* gene and the enhancer signals at the 3′end of *Glis1* could be identified also in human mesenchymal stem cells, but not in other inspected human cell types, suggesting that the complex regulation of *Ahr* and *Glis1* expression in these multipotent cells could be conserved and relevant also in human development (Supplementary Figure S8).

### Overexpression of AHR and GLIS1 can inhibit bone marrow stromal progenitor differentiation

Based on the above results, we reasoned that *Ahr* and *Glis1* could play important roles in maintaining mesenchymal bone marrow progenitors in a multipotent state. Indeed, previous work has separately shown that both adipogenesis and osteoblastogenesis can be inhibited by toxic compounds like dioxin that are xenobiotic ligands of AHR (71–73), while GLIS1 has been shown to promote reprogramming of fibroblasts to induced pluripotent stem cells (iPSCs) (61).

**Figure 6.**
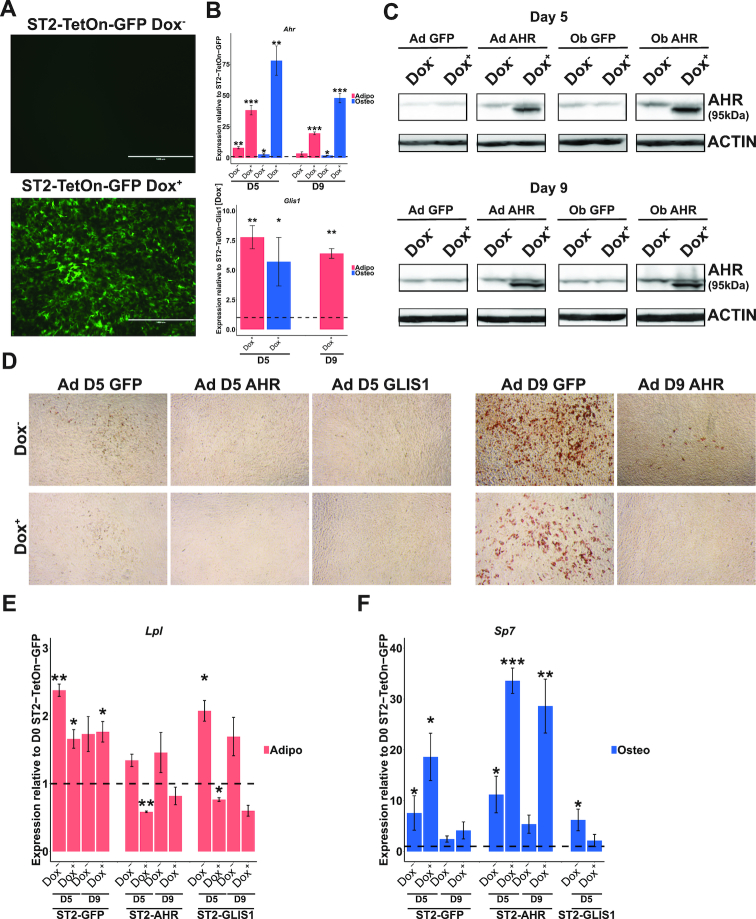
*Ahr* and *Glis1* inhibit the differentiation of multipotent ST2 cells. (**A**–**C**) Establishment of stable inducible AHR and GLIS1 expressing ST2 cell lines. (A) Control cell lines with integrated *CopGFP* gene under a Tet-On 3G promoter (ST2-TetOn-GFP) can be induced for GFP expression by DOX treatment for 24 h. (B) Stable ST2 cell lines with integrated *Ahr* gene (ST2-TetOn-AHR) or *Glis1* (ST2-TetOn-GLIS1) gene under TetOn 3G promoter overexpress AHR and GLIS1, respectively. ST2 cell lines were differentiated for 5 days (D5) or 9 days (D9) toward adipocytes (in magenta) or osteoblasts (in light blue) in the presence or absence doxycycline (Dox^+^ or Dox^−^) and RT-qPCR was performed for *Ahr* and *Glis1* to test for TF overexpression compared to the differentiated ST2-TetOn-GFP cell lines. *Glis1* primers were specific for the inserted codon-optimized *Glis1* sequence and do not detect the endogenous *Glis1* mRNA. Therefore no normalization to the ST2-Tet-On-GFP cells could be performed. The statistical significance for RT-qPCR measurements compared to the value in the similarly differentiated ST2-TetOn-GFP cells (for *Ahr*) or ST2-TetOn-GLIS1 (for *Glis1*) was determined by one sample *t*-test. * = *P*< 0.05, ** = *P*< 0.01 and *** = *P*< 0.001. Data points represent mean of three independent stable cell lines ± SEM. (C) Western blot analysis of AHR expression in ST2-TetOn-AHR cells and ST2-TetOn-GFP cells on D5 and D9 of differentiation toward adipocytes (upper panel) or osteoblasts (lower panel) in presence (Dox^+^) or absence (Dox^−^) of doxycylin. No GLIS1 Western blot was performed due to lack of a specific antibody. (**D**) Inhibition of adipocyte differentiation by overexpression of AHR or GLIS1. Oil Red O staining of the indicated ST2 cell lines on D5 and D9 of differentiation. No staining of ST2-TetOn-GLIS1 cells could be performed on D9 due to decreased adherence of the cells. (**E** and **F**) Changes in (E) adipogenesis and (F) osteoblastogenesis marker genes upon TF overexpression. ST2 cell lines were differentiated for 5 days (D5) or 9 days (D9) toward adipocytes or osteoblasts in the presence or absence of doxycycline (Dox^+^ or Dox^−^) and RT-qPCR was performed for (E) *Lpl* and (F) *Sp7*. The expression in the undifferentiated (D0) ST2-TetOn-GFP cells was set to 1 and is indicated by the dashed line. The statistical significance for RT-qPCR measurements compared to the value in the undifferentiated ST2-TetOn-GFP cells was determined by one sample *t*-test. * = *P*< 0.05, ** = *P*< 0.01 and *** = *P*< 0.001. Data points represent mean of 3 independent stable cell lines ± SEM.

To investigate whether a failure to downregulate AHR or GLIS1 upon differentiation would interfere with the lineage commitment, we generated stable doxycyclin inducible ST2 cell lines capable of overexpressing AHR or GLIS1 (ST2-TetOn-AHR and ST2-TetOn-GLIS1 cells, see ‘Materials and Methods’ section for details). As control cells, inducible CopGFP expressing cell lines were generated (ST2-TetOn-GFP). To test the inducibility of the generated cell lines, the expression of CopGFP was observed via fluorescence microscopy. As shown in Figure 6A, 24 h treatment of the generated ST2-TetOn-GFP cell lines with doxycycline (Dox+) was able to induce high levels of GFP expression.

Next all the generated cell lines were differentiated toward adipocytes or osteoblasts either in presence (Dox+) or absence (Dox^−^) of doxycycline and RNA and protein were collected at D5 or D9 of differentiation. The significantly and strongly induced levels of both *Ahr* and *Glis1* expression could be confirmed by RT-qPCR during differentiation in the respective cell lines under Dox^+^ conditions (Figure 6B). However, for both TFs elevated expression levels of the inserted TFs were also detected in the Dox^−^ conditions, suggesting existing activity of the Tet-On 3G promoter also in the absence of doxycycline. At last, to directly test the increased TF expression at the protein level, western blotting for samples from the same conditions was carried out (Figure 6C). AHR was confirmed to be elevated in ST2-TetOn-AHR cells both in Dox^−^ and Dox^+^ conditions, with a particularly high induction visible in the presence of doxycycline. For GLIS1 none of the available tested antibodies were found to be specific (data not shown).

To directly assess the impact of the AHR and GLIS1 expression on the adipocyte differentiation of the ST2 cells, we performed Oil Red O staining of lipid accumulation on the D5 and D9 of adipogenesis. While ST2-TetOn-GFP control cells showed some accumulation of lipids already on D5, as indicated by the faint red staining, no such staining could be observed in ST2-TetOn-AHR or ST2-TetOn-GLIS1 cells at the same time point (Figure 6D). Moreover, this difference became even clearer on D9 of differentiation when the control cells showed strong red staining of the lipids in both Dox^+^ and Dox^−^ conditions, while in AHR expressing cells only modest lipid accumulation could be observed in Dox^−^ condition, and no accumulation at all in the Dox^+^ condition. Also GLIS1 expressing cells did not show additional lipid accumulation after D5. However, this could not be confirmed by Oil Red O staining as in particular the GLIS1 expressing cells were loosing their adherence along the progression of the differentiation.

To confirm the observed differentiation defects in the presence of high AHR and GLIS1 levels, RT-qPCR analysis of the known adipocyte marker gene *Lpl* was performed. In ST2-TetOn-GFP cells *Lpl* was upregulated by D5 of differentiation and remained elevated in D9 cells both in presence and absence of doxycycline (Figure 6E). However, consistently with the Oil Red O staining results, the ST2-TetOn-AHR cells showed reduced induction already in Dox^−^ conditions and no induction at all upon additional AHR overexpression. Similarly, GLIS1 overexpressing cells failed to induce *Lpl* expression, indicating a failure to differentiate toward adipocytes.

Next, the impact of the two TFs on osteoblast differentiation was investigated by RT-qPCR analysis of the known osteoblast marker gene *Sp7*. Again the ST2-TetOn-GFP cells showed strong upregulation of *Sp7* by D5 of differentiation and remained somewhat elevated on D9 of differentiation (Figure 6F). To our surprise, the presence of the doxycycline in the differentiation culture further increased the *Sp7* induction, indicating that doxycycline alone might influence osteoblast differentiation. Indeed, existing literature indicates a role for tetracyclines as positive regulators of bone formation and osteoblast differentiation via their effects on metalloproteinases and Wnt signaling (74,75). Also in ST2-TetOn-AHR cells the presence of doxycycline was increasing the *Sp7* expression upon induction of differentiation, complicating the conclusion on AHR’s effect on osteoblastogenesis. However, in ST2-TetOn-GLIS1 cells the significant induction of *Sp7* on D5 in the absence of doxycycline was completely lost upon GLIS1 overexpression, indicating that GLIS1 expression inhibits also osteoblast differentiation. Moreover, the GLIS1 overexpressing cells were undergoing increased levels of cell death specifically in osteoblast differentiating cells, preventing the collection of samples after D5 of differentiation (data now shown). Consequently, also von Kossa staining for mineralization of the mature osteoblasts could not be performed with these cells.

### AHR and GLIS1 regulate mesenchymal multipotency through repression of lineage-specific genes

**Figure 7.**
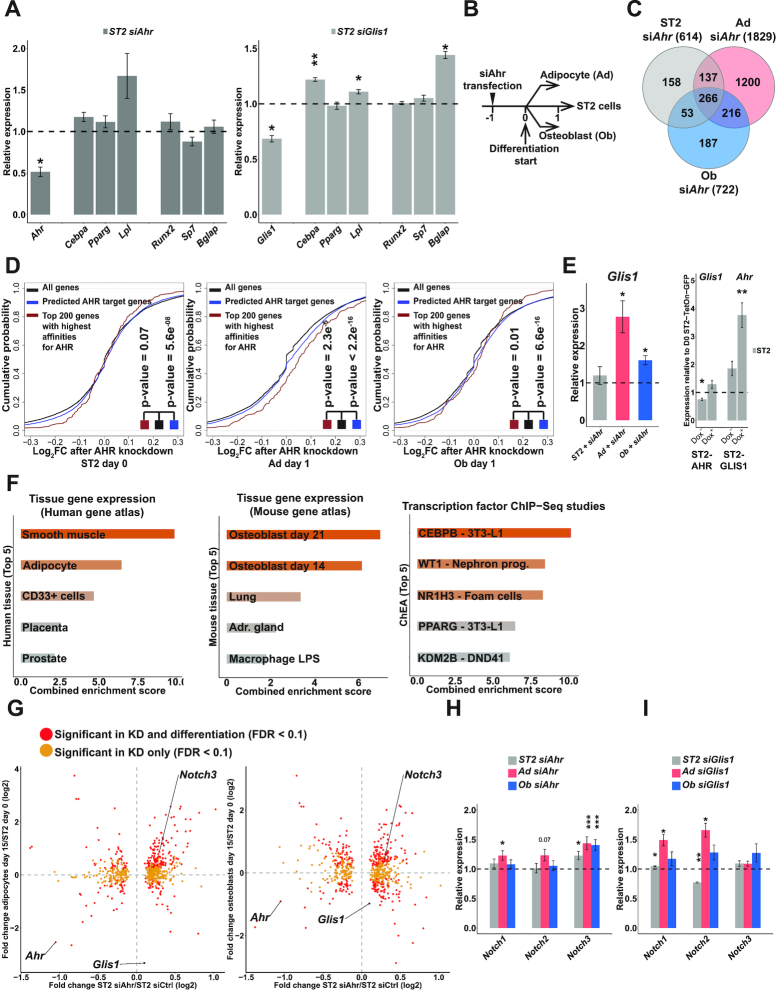
AHR and GLIS1 KDs lead to induction of lineage-specific genes and confirm EPIC-DREM predicted AHR targets. (**A**) Relative expression levels of differentiation marker genes following AHR or GLIS1 KD in undifferentiated ST2 cells are shown (*y*-axis). The statistical significance for RT-qPCR measurements of KD cells against cells transfected with siControl was determined by a two-tailed Student’s *t*-test (* = *P*< 0.05, ** = *P*< 0.01 and *** = *P*< 0.001). Data points represent mean of three biological replicates ± SEM. (**B**) Schematic representation of the KD RNA-seq experiments. See Supplementary Figure S9 for more details on KD efficiency. (**C**) Venn diagram comparing the differentially expressed genes from the three different AHR KD conditions (FDR < 0.1) identified 266 genes consistently deregulated in all conditions. (**D**) Confirmation of EPIC-DREM predictions. Cumulative distribution of all expressed genes (black line), all EPIC-DREM predicted AHR target genes at the corresponding condition (blue line) and top 200 targets of AHR (red line) in relation to their log_2_ FC upon AHR depletion per condition as indicated. The significance of the increased FC of the AHR targets was confirmed with the Kolmogorov–Smirnov test and the corresponding *P*-values are indicated. (**E**) Relative expression levels of *Glis1* mRNA following AHR KD in the three indicated conditions and following AHR overexpression in undifferentiated ST2 cells, and level of *Ahr* following GLIS1 overexpression. See panel 7A and panel 6F for more details on RT-qPCR analysis. (**F**) Enrichment analysis of the 266 AHR targets identified in panel C for their preferred tissue expression profiles in human and mouse, and proteins identified as shared regulators in existing ChIP-seq studies. Top five most enriched hits are shown for each type of enrichment. *X*-axis indicates the combined enrichment score (43) and coloring the enrichment *P*-value. (**G**) Scatter plots indicating the transcriptome-wide log_2_ FCs as measured by RNA-seq in undifferentiated ST2 cells with reduced AHR levels (*x*-axis) in comparison to the log_2_ FCs of the same transcripts in D15 differentiated adipocytes (left panel) or osteoblasts (right panel). The color code separates transcripts expressed differentially only in AHR-KD (yellow), or both in KD and in differentiation (red). (**H**) *Notch3* is induced upon AHR downregulation in all tested conditions, as measured by RNA-seq, while (**I**) *Notch1* and *Notch2* are affected by GLIS1 KD. See panel A for more details on RT-qPCR. See also Supplementary Figure S9 for expression dynamics of *Notch* receptors during differentation.

Given the impact of AHR and GLIS1 overexpression on ST2 differentiation, we wanted to interrogate whether also the endogenous TFs could directly influence the multipotent state of the bone marrow progenitor cells, and what are their target genes. Therefore we performed a knock-down (KD) of the endogenous AHR and GLIS1 in the ST2 cells and tested the expression of lineage-specific marker genes used earlier to confirm differentiation (Figure 7A; Supplementary Figures S9A–C and S1). For AHR a KD of ∼50% could be confirmed at both mRNA and protein level, while *Glis1* reduction was around 30% and could not be confirmed at the protein level due to lack of a specific antibody. Still, GLIS1-KD led to a modest but significant induction of *Cebpa, Lpl* and *Bglap* expression, while AHR-KD affected the *Lpl* marker gene, consistent with the previous results from the overexpression experiments. Thus, both AHR and GLIS1 might contribute to maintenance of the multipotent state.

While AHR is best known for its role as a xenobiotic receptor, it has been recently suggested that it could also play a role in stem cell maintenance in HSCs under the control of endogenous ligands (76). Moreover, since GLIS1-KD was not more robust and could not yet be confirmed on the protein level, we next focused on the AHR-KD. To directly test whether the endogenous activity of AHR is important for the maintenance of the appropriate transcriptional program of the ST2 cells, we knocked down AHR in undifferentiated cells, and those differentiated for one day toward either lineage (Figure 7B and Supplementary Figure S9B). Two days following the KD total RNA was extracted and subjected to RNA-seq analysis.

The number of genes affected by the AHR-KD (FDR ≤ 0.1) compared to the respective control siRNA transfections was greatly dependent on the cellular condition and ranged from 614 genes in the undifferentiated cells to 722 and 1819 genes in the one day differentiated osteoblasts and adipocytes, respectively (Figure 7C and Supplementary Table S9). The higher extent of genes affected in the differentiated cells is well in keeping with the number of genes normally changing in the early osteoblasto- or adipogenesis, with adipogenesis associated with more changes (Figure 1). This suggests that early changes induced by the knock-down in the undifferentiated cells are amplified in the differentiating cells.

At first, we took advantage of these context-specific KD data to ask whether the EPIC-DREM predicted primary AHR targets at the corresponding time points were indeed affected by depletion of AHR. As shown in Figure 7D, at each condition the predicted AHR targets were significantly more affected by AHR-KD than all genes on average. Especially the top genes with highest affinity scores for AHR regulation on day 1 of adipogenesis were clearly shifted toward more upregulation at each condition (to the right in the cumulative distribution plot), arguing for functional relevance of the EPIC-DREM predictions and for AHR’s role as transcriptional repressor. As highlighted earlier in Figure 3A, AHR was predicted by EPIC-DREM to act as a direct regulator of *Glis1* in undifferentiated cells and during early adipogenesis. To test this specific prediction we performed RT-qPCR for *Glis1* in all AHR-KD samples and confirmed a significant change in *Glis1* levels upon AHR depletion in both differentiating adipocytes and osteoblasts (Figure 7E, left panel). However, AHR overexpression failed to induce consistent effects onto *Glis1* levels in the undifferentiated ST2-TetOn-AHR cells (Figure 7E, right panel). Interestingly, GLIS1 overexpression did induce *Ahr* levels in the undifferentiated ST2-TetOn-GLIS1 cells (Figure 7E, right panel), thereby suggesting existence of feedback regulatory loops that will require further analysis.

To better understand the function of the putative AHR target genes in the ST2 cells, we overlapped the differentially expressed genes from the undifferentiated cells with those identified in the two other KD experiments and obtained 266 high-confidence target genes that were affected in all three conditions (Figure 7C). Enrichment analysis for tissue-specific expression profiles of these genes in human and mouse gene atlas databases revealed smooth muscle, adipocytes and osteoblasts as the most enriched cell types, where the AHR regulated genes are normally expressed (Figure 7F). Consistently, inspection of publicly available ChIP-seq data from various cell types revealed the other regulators of the AHR targets to include CEBPB, PPARG, and NR1H3, all of which are important regulators involved in induction of genes during adipocyte differentiation (77,78).

To further elucidate the role of AHR in the undifferentiated cells and the potential impact on lineage-specific genes, we asked how the deregulated genes were expressed in normally differentiated day 15 adipocytes and osteoblasts. The scatter plots in Figure 7G indicate the transcriptome changes taking place upon AHR-KD in ST2 cells compared to changes of the same genes in the differentiated cell types. As indicated by the color coding, approximately half of the genes affected by AHR-KD were also differentially expressed after normal differentiation (286 in adipocytes and 314 in osteoblasts). Moreover, genes that were upregulated in AHR-KD displayed preferential induction in the differentiated cell types (Figure 7G, upper right quartile). Therefore, AHR might serve as a guardian of the multipotent state in the undifferentiated cells, with its downregulation allowing increased expression of the lineage-specific genes.

Taken together, the above findings support a role for AHR as a regulator of lineage-specific genes that need to remain repressed in the multipotent bone marrow progenitor cells. Among such genes induced upon AHR-KD we identified *Notch3*, a known regulator of cellular differentiation (79) (Figure 7G and H). In both differentiation time courses *Notch3* expression showed an anti-correlated profile compared to *Ahr*, with *Notch3* becoming induced while *Ahr* levels decreased (Figure 5; Supplementary Figure S9D and E). The *Notch3* induction was also accompanied by increased *Notch4* levels in both lineages while the third abundantly expressed receptor, *Notch1*, was concomitantly downregulated (Supplementary Figure S9D and E). At last, to see whether the impact of AHR on *Notch3* is mediated via GLIS1, we tested by RT-qPCR the effect of GLIS1-KD on three of the *Notch* genes in the corresponding conditions (Figure 7I). Interestingly, no effect could be seen on *Notch3* expression upon GLIS1-KD while both *Notch1* and *Notch2* were modestly affected. Thus, it appears that Notch signaling could be a downstream target of AHR and GLIS1 in the commitment of the mesenchymal bone marrow progenitors.

## DISCUSSION

The ability to obtain unbiased GRNs and to identify their key nodes for any given cell state transition in a data-driven manner is becoming increasingly relevant for regenerative and personalized medicine. Understanding such dynamic networks can be improved by obtaining genome-wide time-series datasets such as transcriptomics or epigenomics data. To seamlessly integrate such datasets we have combined time point-specific high accuracy TF binding predictions with probabilistic modeling of temporal gene expression data and applied it to our own time-series data from mesenchymal differentiation (Figures 2 and 3). Similar time series data collections have previously been used to study for example hematopoiesis (7) and myeloid differentiation (6). However, the derived dynamic GRNs have relied on experimentally identified TF binding sites covering only a fraction of all TF–target gene interactions, or on a sub-network of selected TF–TF interactions, respectively.

EPIC-DREM can reveal the key TFs controlling co-expressed gene sets of interest. Still, consistent with the co-operative nature of TF activity (11), the number of putative master regulators is often very large. Recent work elucidating the role of SEs in controlling cell type-specific master regulators has provided researchers with a new tool for data-driven identification of such regulators (9,10). We hypothesized that merged SEs with dynamic behavior during differentiation based on their H3K27ac signal would allow finding the genes, including the TFs, most relevant for the dynamic process. Indeed, quantification of merged SEs shows high correlation with expression levels of their putative target genes over time, both validating the approach and allowing for more accurate association of SEs to their target genes (Figure 4). A recent study applied a similar strategy for SE quantification and further showed that SE dynamics, as measured by MED1 occupancy, were predictive of enhancer looping to target genes, and highlighted H3K27ac as the histone modification that best predicted such loop dynamics, further supporting the validity of our approach (80).

Combining EPIC-DREM results and dynamic SE profiling points toward several TFs with potentially significant roles in both lineages, including *Foxn1, Ahr* and *Glis1* (Figure 4). In addition, dynamic SE profiling supports a role for *Hoxa10*, although EPIC-DREM analysis did not place it among the top TFs. Both *Ahr* and *Glis1*, and their SEs, show an overall reduction in signal during both differentiations, although with differential and lineage-specific dynamics (Figure 5 and Supplementary Figure S6), suggesting that they could play an important role in maintaining multipotent cells. On the contrary, the SE at the *Foxn1* locus shows a decrease in its signal in adipocyte differentiation (data not shown), while in osteoblast differentiation the signal is further increased. Thus suggesting a role as a positive driver of osteoblast differentiation, while potentially having a negative role in adipocytes. Interestingly, FOXN1 has not been previously shown to be involved in mesenchymal bone marrow progenitor differentiation and our results warrant a further investigation of FOXN1 function in adipocyte and osteoblast differentiation.

Also GLIS1 has not been functionally associated to adipocyte or osteoblast differentiation although recent work has implicated it as differentially expressed in brown adipocyte differentiation (81). However, consistent with a potential role in the multipotent progenitors, GLIS1 has been shown capable of promoting reprogramming of fibroblasts to induced pluripotent stem cells (iPSCs) (61). Interestingly, our analysis for enhancer signals at the *GLIS1* locus in different human cell types confirmed the presence of active enhancers also in human mesenchymal stem cells but not in embryonic stem cells (ESCs) (Supplementary Figure S8). Thus, the ability of GLIS1 to promote cellular reprogramming of iPSCs might reflect its endogenous functions in multipotent stem cells like mesenchymal stem cells, rather than the pluripotent ESCs. Our results implicate GLIS1 as a regulator of multipotent ST2 cells with its reduction influencing several lineage-specific genes and a failure to downregulate GLIS1 upon differentiation preventing normal lineage commitment (Figures 6 and 7). In addition, both network analysis and the expression dynamics suggest a role for GLIS1 during adipogenesis (Figure 3A and Supplementary Figure S6). Further work will be needed to elucidate the exact role of GLIS1 in bone marrow and adipocyte differentiation.

Unlike for GLIS1 and FOXN1, previous work has already linked AHR separately to inhibition of both adipocyte and osteoblast differentiation through studies on biological impact of dioxin, an environmental toxin capable of activating AHR (71–73). In 3T3-L1 adipocytes this inhibition is known to be mediated through overexpression of *Ahr* in a dioxin-independent manner (82), while increased levels of *Ahr* expression in mesenchymal stem cells in rheumatoid arthritis are inhibitory of osteogenesis (83). Our results confirm the inhibitory effect of AHR overexpression on differentiation of bone marrow adipocytes while the effect of AHR expression alone on osteoblastogenesis remains unclear (Figure 6). This could be related to the differential dynamics of *Ahr* in the two lineages, with *Ahr* showing induction in early osteoblastogenesis prior to its repression at later time points. Very recent work from Watson *et al.* (84) demonstrated the inhibition of osteoblast differentiation and the persistence of a multipotent state in human bone marrow mesenchymal stem cells upon AHR ligand activation. Future work will be needed to reveal whether ligand availability and AHR transactivation status influence its effects of osteoblastogenesis.

Nevertheless, the exact biological function of AHR in the mesenchymal stem cells has remained unclear while maintenance of HSCs, located in the same niche of the bone marrow, has been suggested to depend on the normal function of AHR (76). Moreover, as HSC maintenance also depends on the cytokines and chemokines provided by the mesenchymal stem cells, AHR is likely to impact HSCs through its gene regulatory functions in both mesenchymal stem cells and HSCs (16,85–86).

Here we show that repression of *Ahr* in mesenchymal differentiation happens with lineage-specific dynamics and is accompanied by similar reduction in signal of an exceptionally large SE cluster downstream of *Ahr*. Together *Ahr* and the SEs form their own TAD in mouse cells and in human cells the SE signal is specific for mesenchymal stem cells. Analyzing the contributions of the different constituents of the *Ahr*-SEs will be important for understanding which pathways converge to regulate *Ahr* in mesenchymal stem cells and whether they function in a synergistic manner as suggested for some other large SEs (87,88).

A KD of AHR expression in the undifferentiated and early stage differentiated cells already confirmed many of the EPIC-DREM predictions and also revealed an enrichment for lineage-specific genes among AHR targets, including *Notch3* (Figure 6). Notch signaling has been implicated in numerous developmental processes with highly diverse outcomes (79) and also in ST2 cells *Notch3* regulation is accompanied by changes in other *Notch* genes (Supplementary Figure S9). Interestingly, AHR has been recently linked to regulation of Notch signaling in mouse lymphoid cells and testis (89,90). However, the affected Notch receptors varied depending on the cell type in question. *Notch* genes show different expression profiles across tissues and cell types and *Ahr*-mediated regulation of Notch signaling could be context-specific depending on the prevailing GRN or chromatin landscape. Curiously, *Notch3* has a cell type-selective expression profile, favoring mesenchymal tissues like bone, muscle and adipose tissue (91).

Our approach for identification of the dynamic GRNs and SEs allows key regulator identification in various time series experiments involving cell state changes. Our current results together with previous data identify AHR and GLIS1 as likely guardians of mesenchymal multipotency, implicate additional TFs as novel regulators of adipocyte and osteoblast differentiation, and provide an extensive resource for further analyses of mesenchymal lineage commitment.

## DATA AVAILABILITY

The datasets generated and analyzed during the current study are available in the European Nucleotide Archive with the accession number PRJEB20933. The ChIP-seq tracks are available at the UCSC Genome Browser (http://genome.ucsc.edu/cgi-bin/hgTracks?hubUrl=https://biostat2.uni.lu/dgerard/hub.txt&genome=mm10). The ChIP-seq tracks from primary bone marrow-derived mesenchymal stem cells published by Meyer *et al.* (18) used for comparative analysis are also available at the UCSC Genome Browser (http://genome.ucsc.edu/cgi-bin/hgGateway?hgsid=229558738_fmRhqQwsRiX7BTTUHjnu7U7vm3Rl).

All scripts and generated GRNs can be retrieved from https://github.com/sysbiolux and from https://github.com/SchulzLab/TEPIC.

## Supplementary Material

Supplementary Data
