# Supplementary material for: Temporal enhancer profiling of parallel lineages identifies AHR and GLIS1 as regulators of mesenchymal multipotency
Source: Nucleic Acids Res. 2018 Dec 14;47(3):1141–63. doi: 10.1093/nar/gky1240 (PMC6380961; doi:10.1093/nar/gky1240)
Supplement: Supplementary Data [file gky1240_supplemental_files.zip › Gerard_et_al_NAR_SUPPLEMENT_REVISED.pdf]

Temporal enhancer profiling of parallel lineages identifies AHR and GLIS1 as regulators of mesenchymal multipotency

Deborah Gérard, Florian Schmidt, Aurélien Ginolhac, Martine Schmitz, Rashi Halder, Peter Ebert, Marcel H. Schulz, Thomas Sauter and Lasse Sinkkonen

Supplementary Information

Supplementary Figures

Supplementary Figure S1: Differentiation of ST2 bone marrow stroma progenitors towards adipocytes and osteoblasts. (A-C) Prior to high-throughput sequencing, the differentiation of ST2 cells towards adipocytes and osteoblasts was controlled using RT-qPCR analysis of the known marker genes of both differentiations and by visual inspection of cell morphology and stainings. Time-series RT-qPCR profiles of three adipocyte markers genes (*Pparg*, *Cebpa* and *Lpl*) and three osteoblast markers genes (*Runx2*, *Sp7* and *Bglap*) are shown for (A) adipocyte differentiation and (B) osteoblast differentiation. The statistical significance for RT-qPCR measurements compared to the value on D0 was determined by two-tailed Student's t-test. *=$p<0.05$, **=$p<0.01$ and ***=$p<0.001$. Data points represent mean of 3 biological replicates +/- SEM. (C) Representative images of undifferentiated ST2 cells and 15 days differentiated adipocytes or 28 days differentiated osteoblasts stained with Oil Red O or Van Kossa, respectively, show morphological differences.

Supplementary Figure S2: **Overview of TF annotation evaluation on ENCODE data.** (A) The plot shows the mean performance of Recall and Precision of EPIC predictions over 78 different TFs as measured on three ENCODE cell lines. Different p-value thresholds are colored and standard error bars are shown. (B) Detailed plots of Precision-Recall analysis of EPIC TF predictions on 3 ENCODE cell lines (rows) for different p-value thresholds and the different representative TFs (columns).

Supplementary Figure S3: PPARG-centric EPIC-DREM network on day 3 of adipogenesis. The upper part shows all transcriptional regulators predicted to target *Pparg* gene on day 3 of adipogenesis by EPIC-DREM based on the time point specific RNA-seq and enhancer ChIP-seq data (H3K27ac). Lower part shows the top 200 targets with highest affinity score for PPARG:RXRA heterodimer binding at the same time point. The list of all 3405 target genes can be found in Supplementary Table S6. Targets and TFs colored in blue are upregulated while those in orange are downregulated compared to the preceding time point. Grey indicates no change in expression.

Supplementary Figure S4: PPARG is regulated by SEs with lineage-specific dynamics in cell lines of white and bone marrow adipocytes. (A) Overview depicting the enrichments of H3K4me3 (in dark blue), H3K36me3 (in green), and H3K27ac (in magenta) at the *Pparg* locus across the time points of adipogenesis of MSCs (ST2 cells) (A) and 3T3-L1 cells (B), respectively. The magenta bars indicate the merged SE regions identified through the analysis described in Figure 4. ST2 cell show high transcriptional activity already in undifferentiated cells while in 3T3-L1 cells the locus and especially the transcript variant 2 are activated only after differentiation initiation. ChIP-seq data for 3T3-L1 cells was published by Mikkelsen *et al*.

Supplementary Figure S5: The profiles of the dynamic merged SEs. (A-B) The dynamic profiles of merged SEs identified by STEM clustering that did not fit (A) the four main clusters identified in adipocytes and (B) the two main clusters identified in osteoblasts in Figure 4.

Supplementary Figure S6. *Glis1* is regulated by a SE with lineage-specific dynamics. (A-B) Overview depicting the enrichments of H3K4me3 (in dark blue and purple), H3K36me3 (in light and dark green), and H3K27ac (magenta and light blue) at the *Glis1* locus across the time points of adipogenesis (A) and osteoblastogenesis (B), respectively. The magenta and light blue bars indicate the merged SE regions identified through the analysis described in Figure 4. See also Supplementary Figure S7. (C-D) *Glis1* downregulation correlates with the signal from SE_{831}. The *Glis1* mRNA level was measured across the differentiation by RNA-seq (upper panel) and RT-qPCR (lower panel) in both adipocyte (C) and osteoblast (D) differentiation and is indicated as the intact line. Dashed line represents the signal from the SE_{831}. r = Pearson correlation co-efficient. The statistical significance for RT-qPCR measurements compared to the value on day 0 was determined by two-tailed Student's t-test. *=$p<0.05$, **=$p<0.01$ and ***=$p<0.001$. Data points represent mean of 3 biological replicates +/- SEM. AD9 sample for H3K27ac and OD15 for H3K4me3 were not included in the above analysis due to lower number of mappable high quality reads. (E) Overview depicting the enrichment of H3K27ac at the *Glis1* locus in the confluent undifferentiated (D0) and differentiated (D7) 3T3-L1 adipocyte cell line. No SE formation could be detected in these more lineage-committed cells. The data were obtained from (Mikkelsen et al., 2010). The H3K27ac enrichments at the corresponding locus in human cell types are indicated in Supplementary Figure S8B.

Supplementary Figure S7: Dynamic SEs correlating with *Ahr* and *Glis1* are located in the same topological domain (TAD) in multiple cell types. Hi-C maps of long-range chromosomal interactions from three unrelated mouse cell/tissue types [(A) CH12 cell line, (B) embryonic stem (ES) cells, and (C) cortex] show high level of similarity between the TAD formation across the

cell types at the *Ahr* locus. *Snx13* and *Ahr*, the two genes flanking the identified merged SEs shown in Figure 5, are located in separate TADs (indicated by the blue and yellow bars, respectively) in all observed cell types with all 4 SEs remaining in the same TAD with *Ahr*. Similarly, SE at the *Glis1* locus remains always in the same TAD with *Glis1* TSS. The Hi-C images were obtained with the 3D Genome Browser[1] (http://www.3dgenome.org.) using data from Rao[2] et al. and Dixon[3] et al..

Supplementary Figure S8: High enhancer signals at *AHR* and *GLIS1* loci are conserved in humans and detected selectively in human bone marrow mesenchymal stem cells. ChIP-seq enrichments of H3K27ac at the *AHR* and *GLIS1* loci in 9 different human cell types[4-10]. Three biological replicates of human bone marrow mesenchymal stem cells[4] (MSCs) show specific and conserved enrichment of H3K27ac at (A) the genomic region flanked by *AHR* and *SNX13*, corresponding to the identified merged SEs shown in Figure 5, and (B) at the 3'end of *GLIS1* gene, corresponding to the identified merged SE shown in Supplementary Figure S6. No corresponding enrichments could be observed in erythroblasts[5], B lymphocytes[6], dendritic cells[7], hematopoietic stem cells[4] (HPSs), natural killer cells[8] (NKCs), ES cells[4] (ESCs), aorta[9], or psoas muscle[9]. All reads were mapped to hg38 and visualized using Integrated Genome Viewer[11, 12].

Supplementary Figure S9: Efficiency of AHR and GLIS1 KD and rewiring of Notch receptor signaling during adipocyte and osteoblast differentiation. (A-B) RT-qPCR and (C) Western blotting were used to confirm downregulation at the mRNA (for *Ahr* and *Glis1*) and protein level (for AHR) when compared to non-transfected (NT) cells or to cells similarly transfected with

unspecific siControl. A representative gel image of undifferentiated ST2 cells and 1 day differentiated adipocytes (Ad) and Osteoblasts (Ob) is shown. The statistical significance for RT-qPCR measurements compared to the value of cells transfected with siControl was determined by two-tailed Student's t-test. *=$p<0.05$. Data points represent mean of 3 biological replicates +/- SEM. The mRNA levels of *Notch1*, *Notch2*, *Notch3*, and *Notch4* as measured by RNA-seq are depicted across the time-series of (D) adipocyte and (E) osteoblast differentiation. The values for all biological replicates are indicated separately.

Supplementary Figure S1

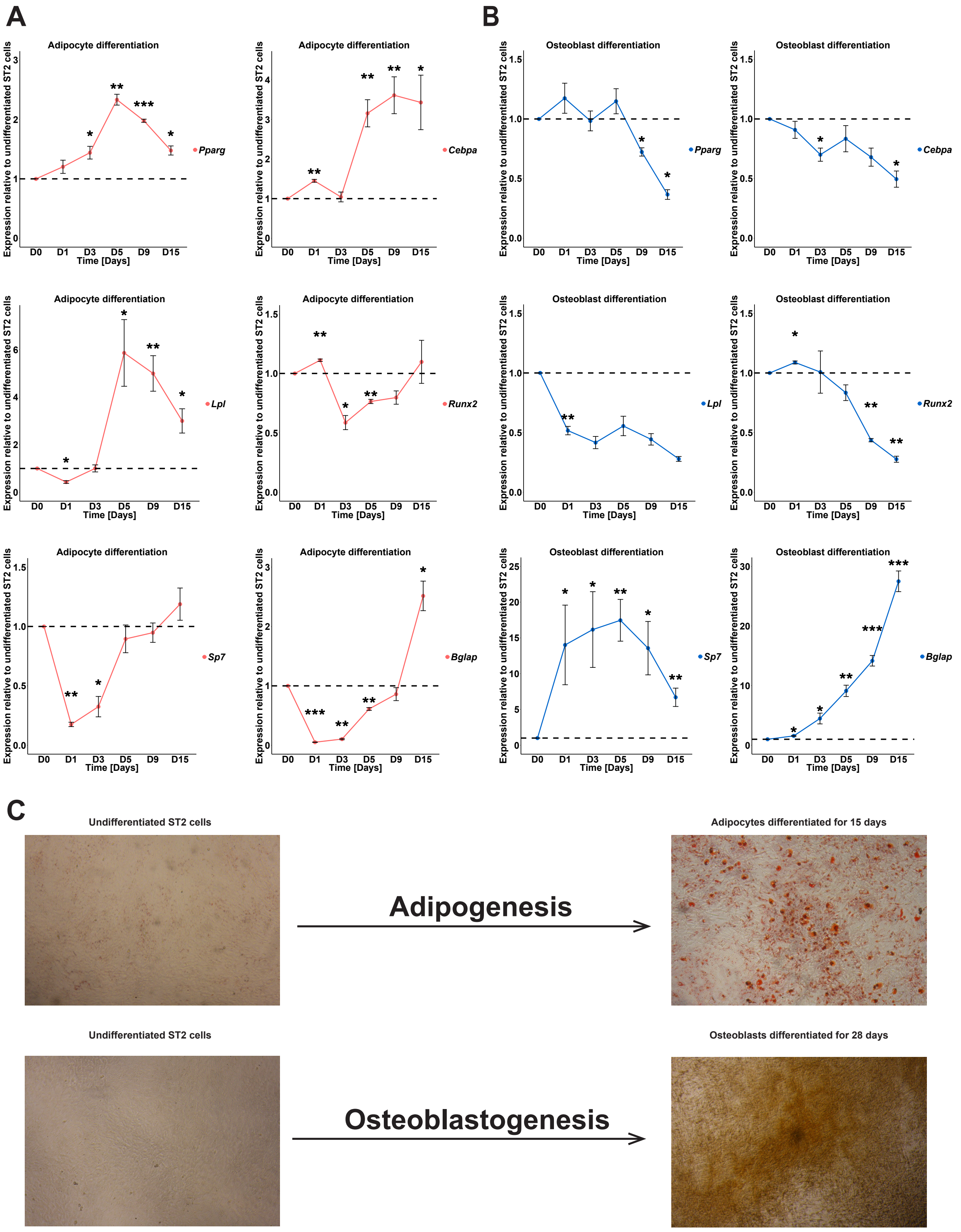

Supplementary Figure S2

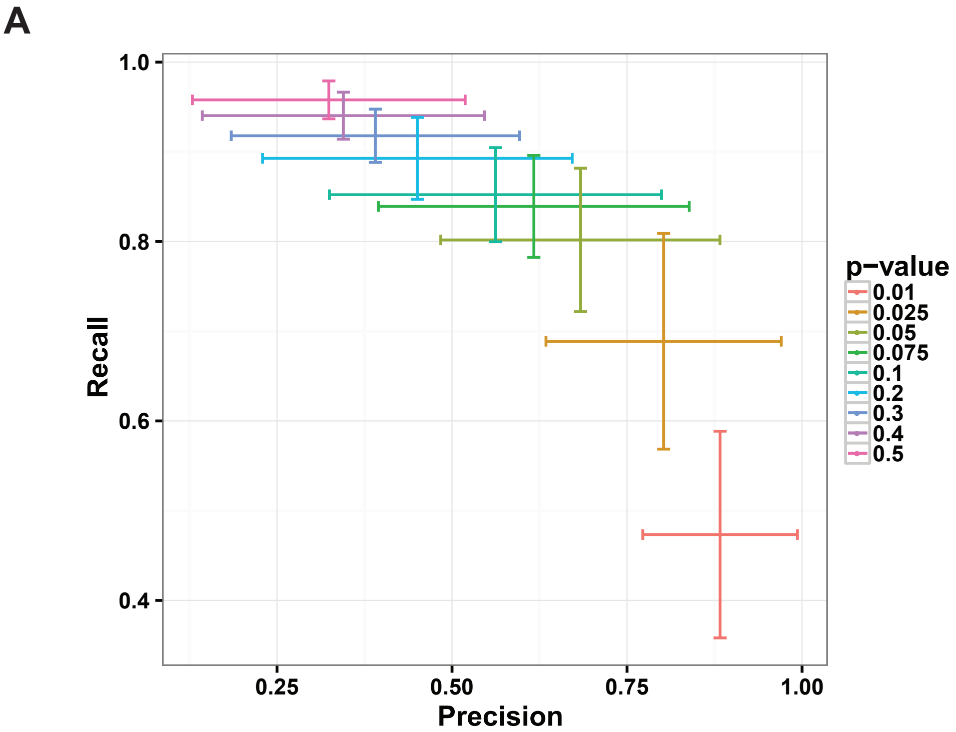

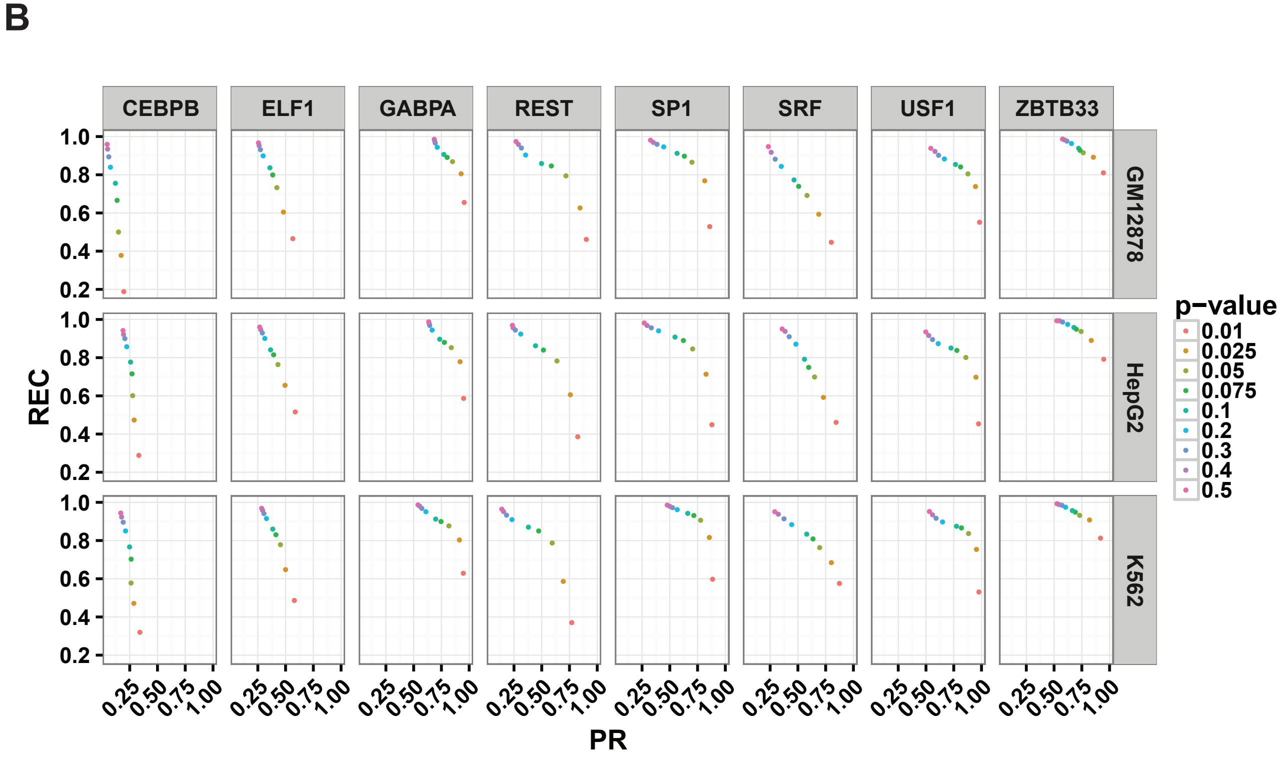

Supplementary Figure S3

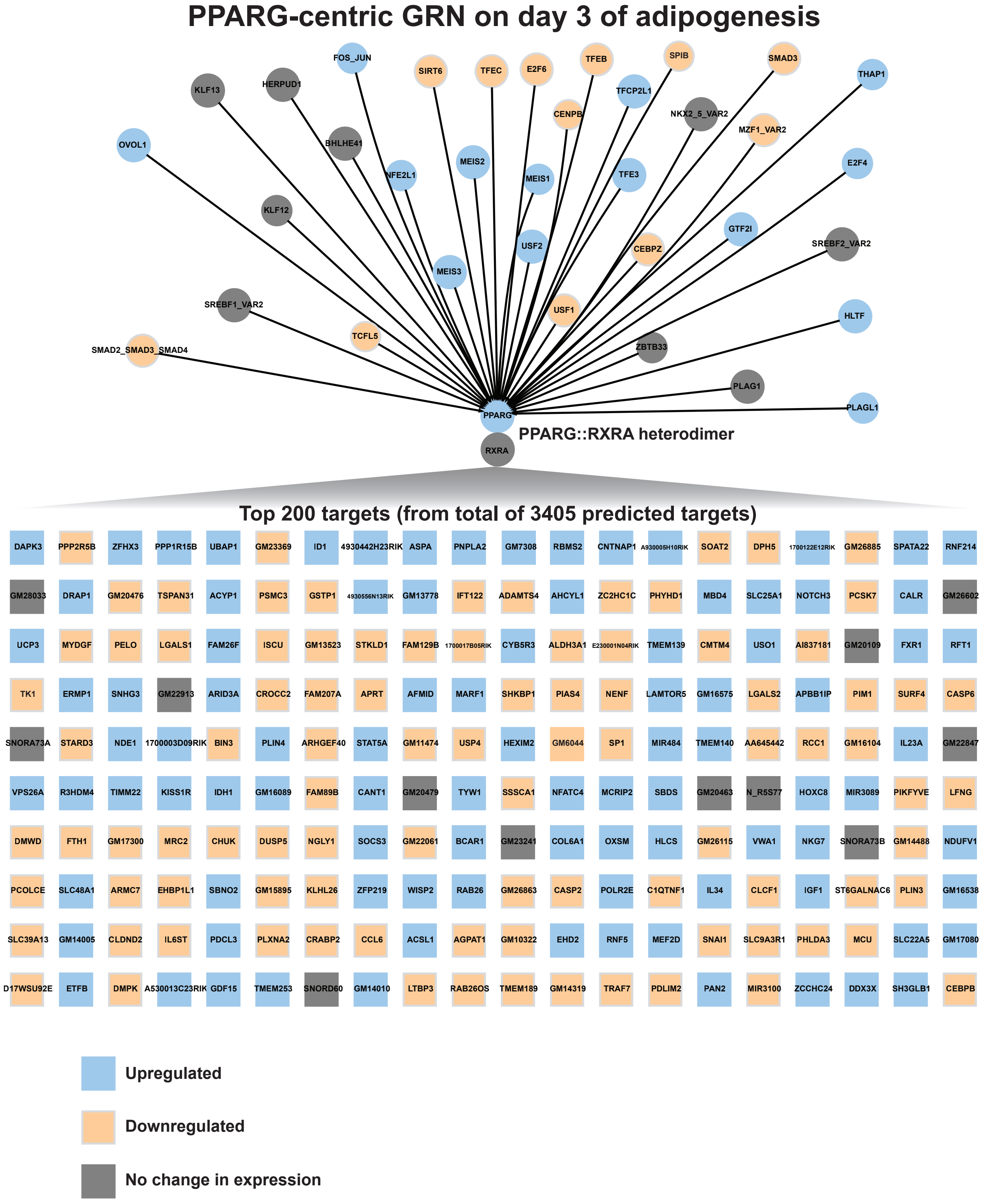

Supplementary Figure S4

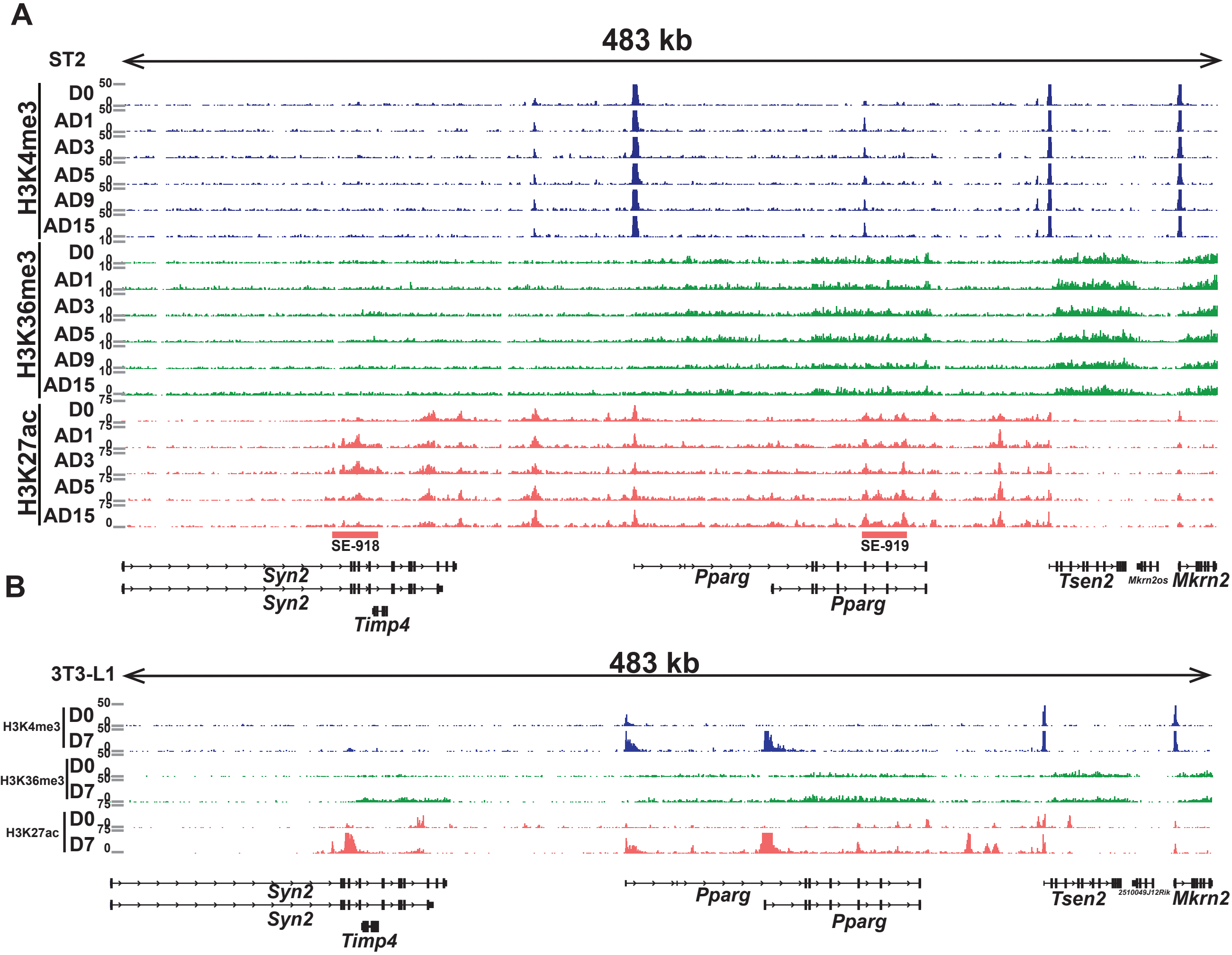

Supplementary Figure S5

A

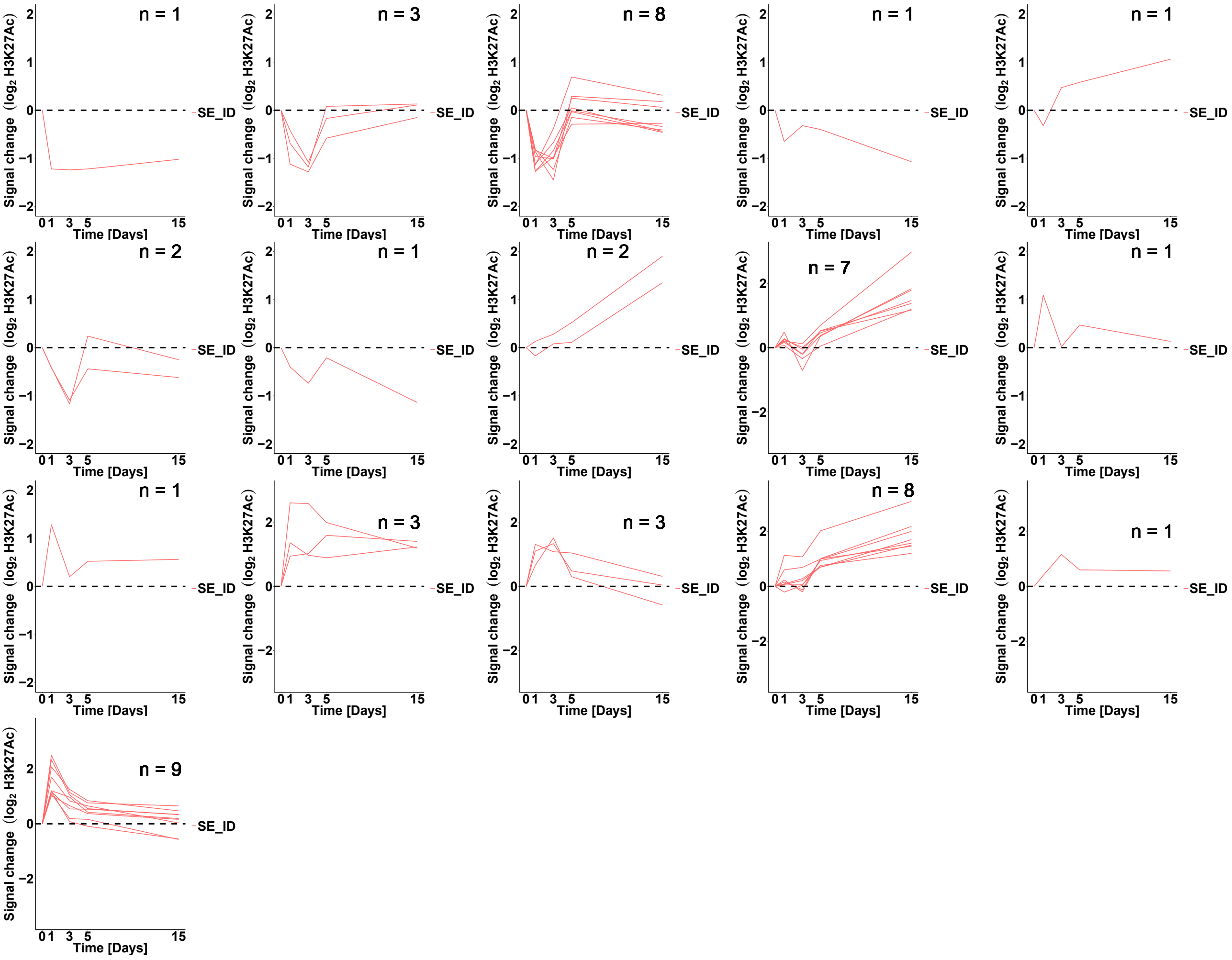

B

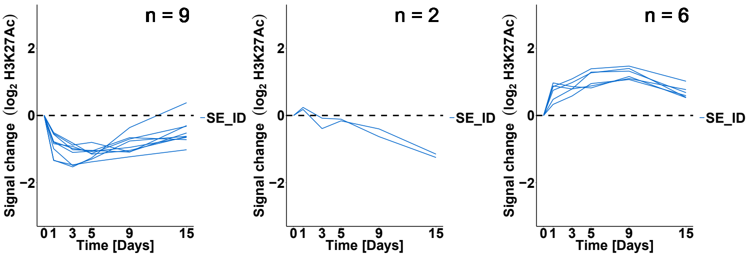

Supplementary Figure S6

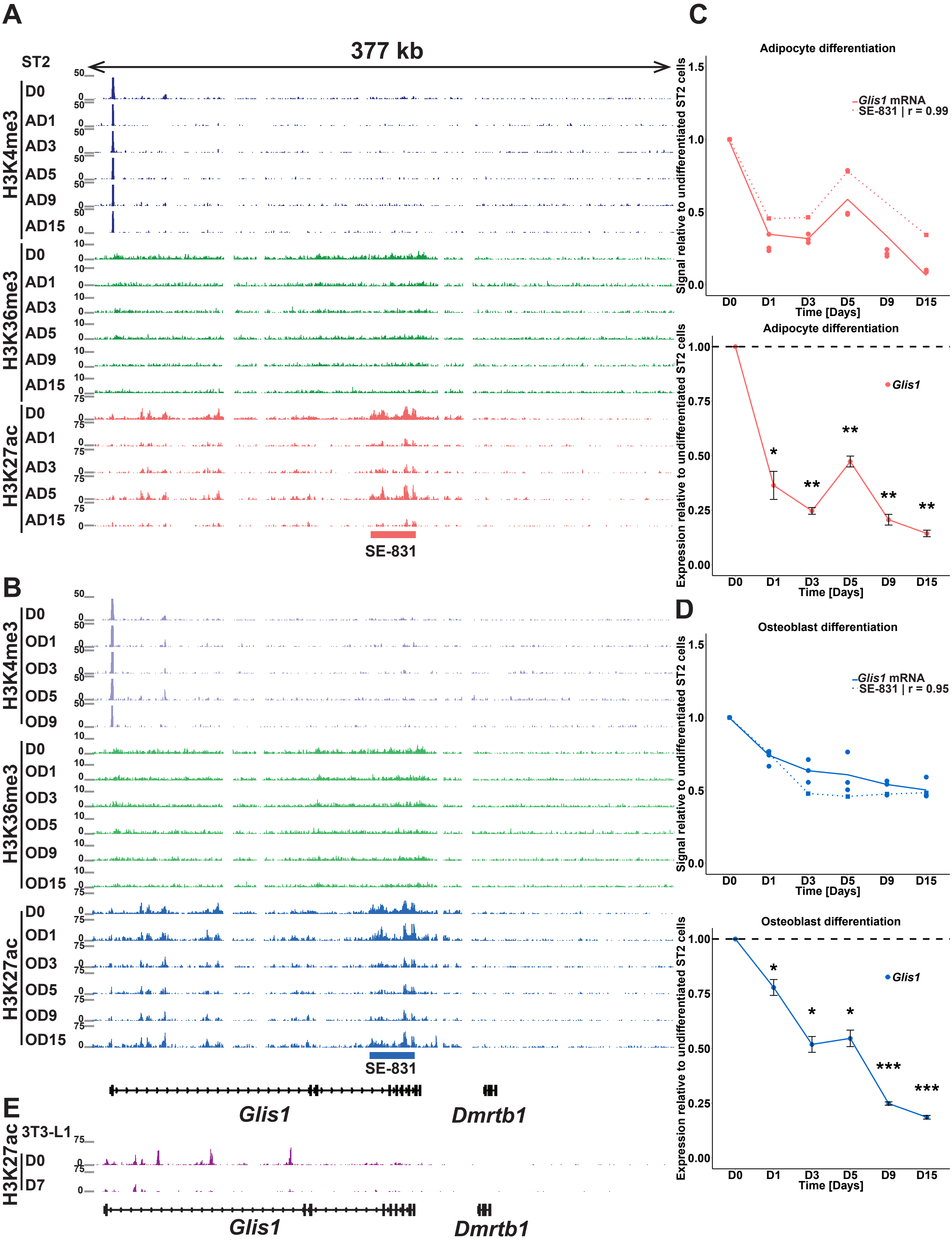

Supplementary Figure S7

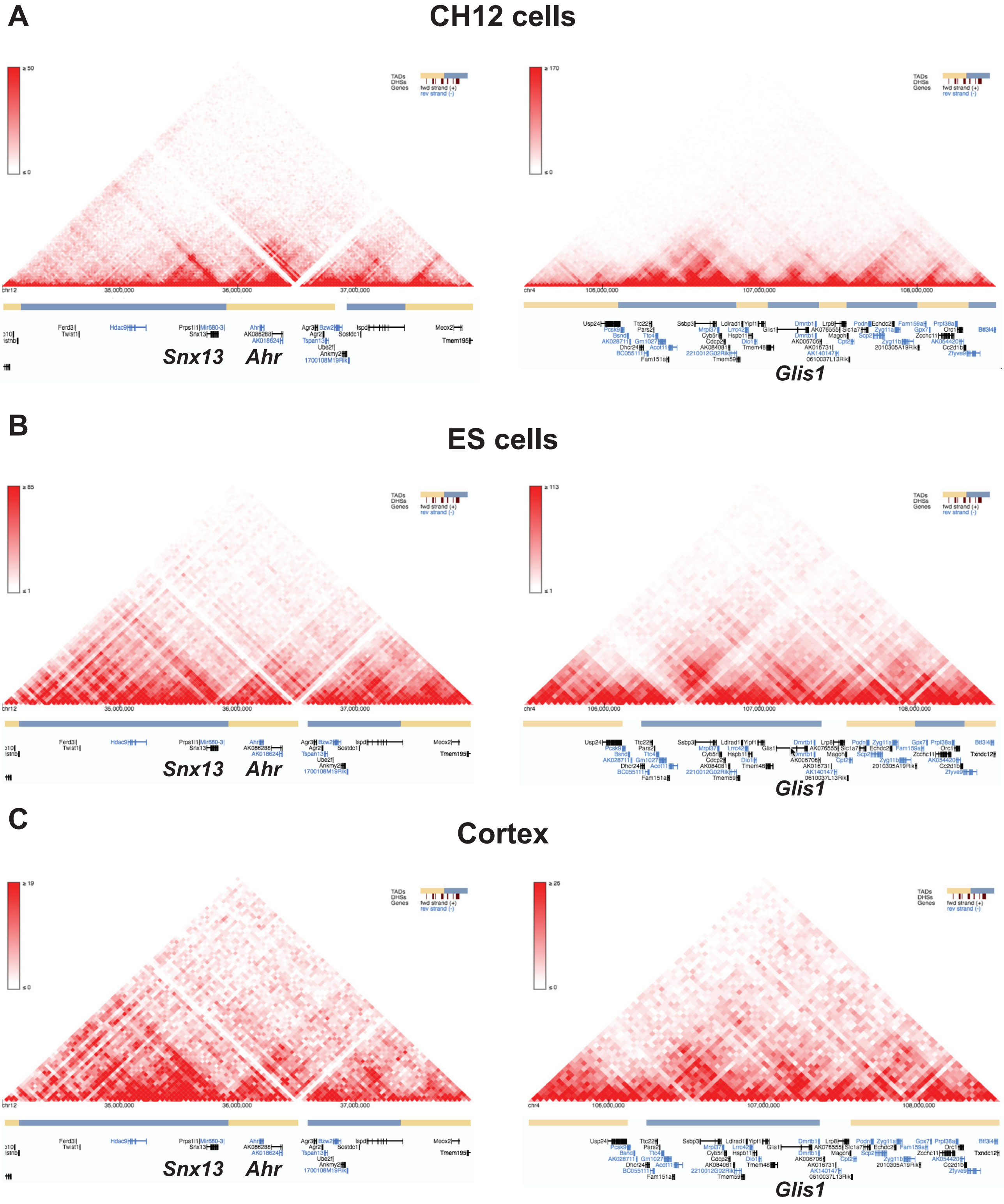

Supplementary Figure S8

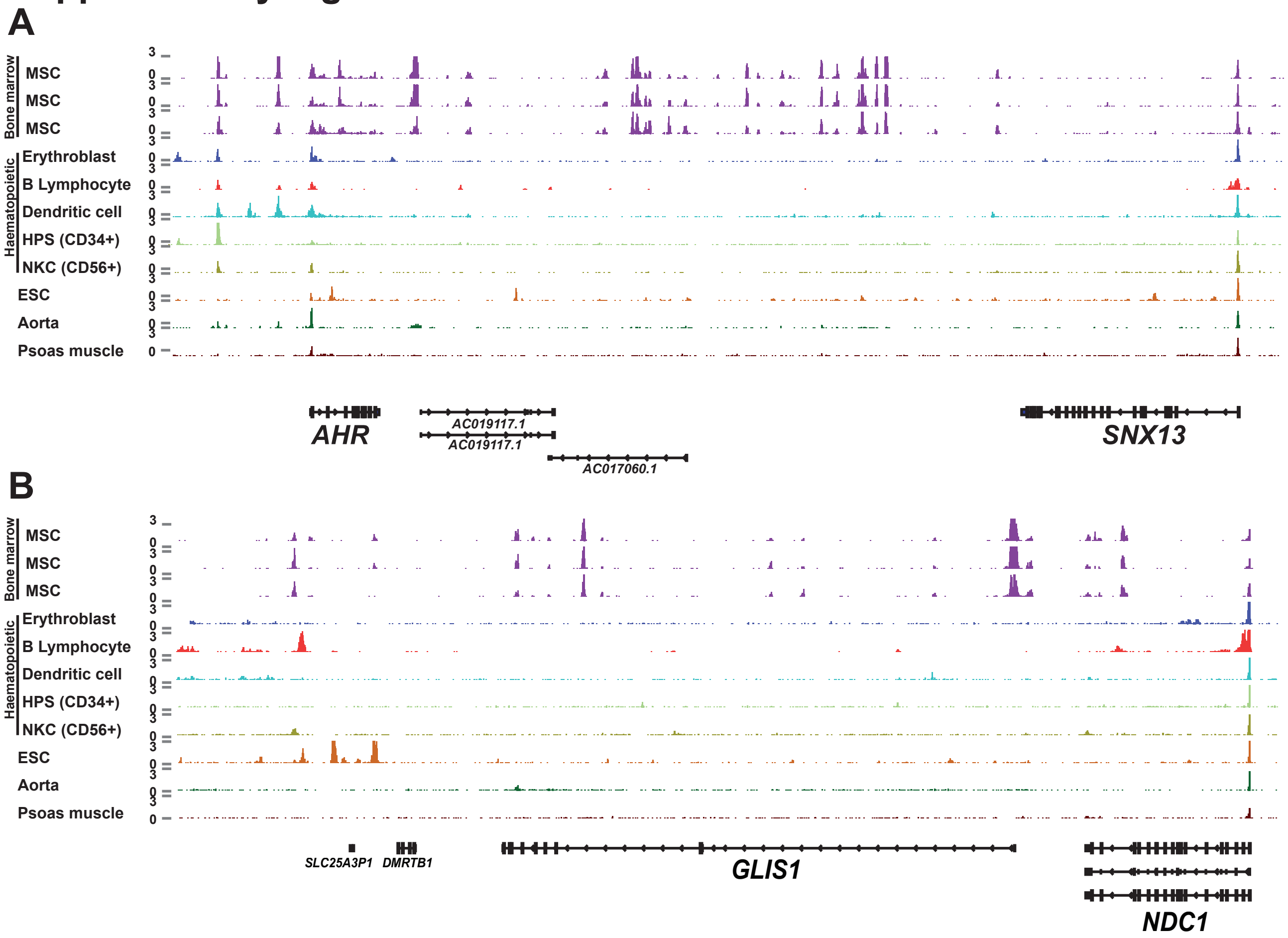

Supplementary Figure S9

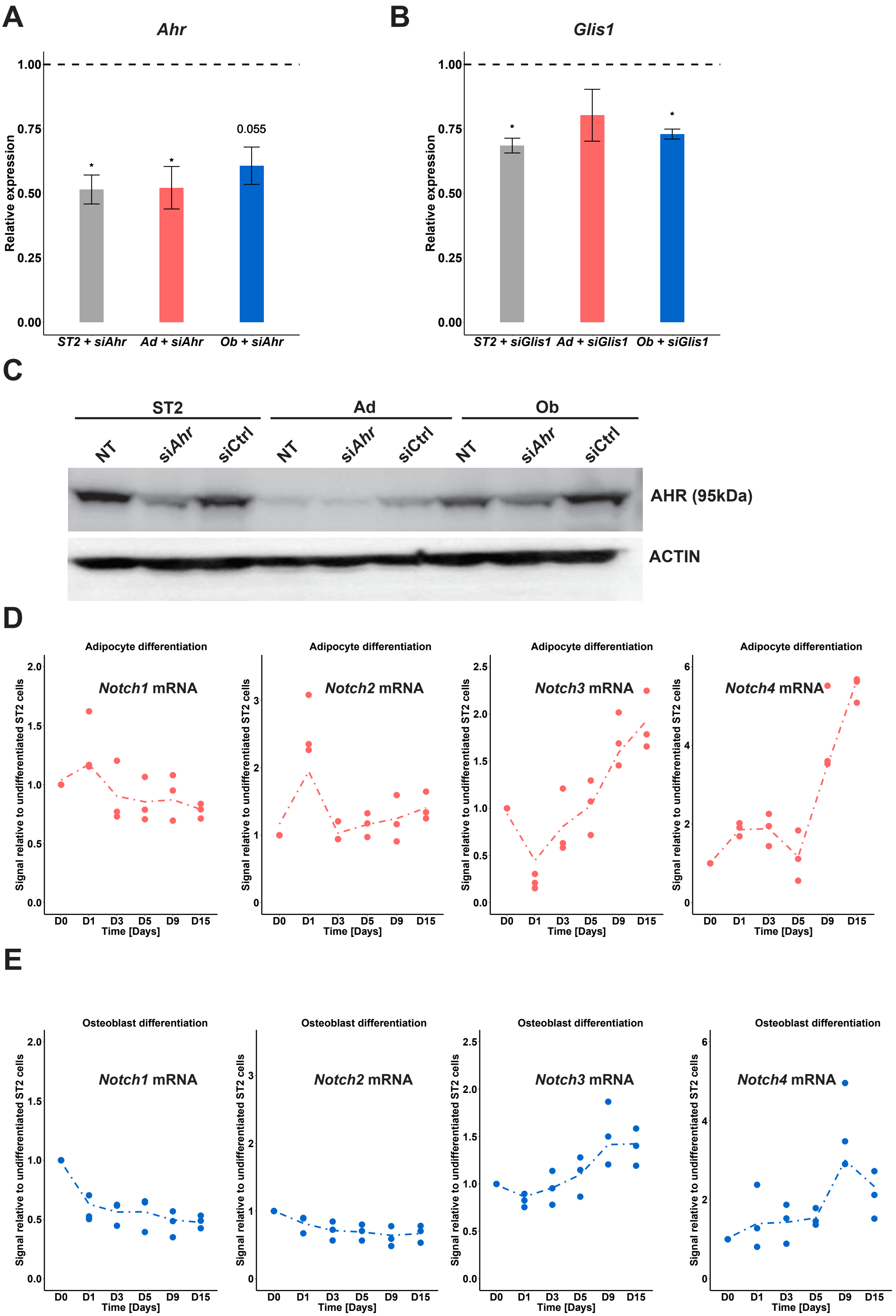

Supplementary Tables

Supplementary Table S1. siRNA and primer sequences used in the study.

siRNA	Sequence (5‘ – 3‘)
si*Ahr*	CCAAUGCACGCUUGAUUUA
	GAAGGAGAGUUCUUGUUAC
	CCGCAAGAUGUUAUUAAUA
	CCAGUUCUCUUAUGAGUGC
si*Glis1*	GCUACAAGCCCUUCAAUGC
	GUUCGAAGGCUGCAGUAAA
	GCUCUAGUUAGCUGUGUAA
	GCUACGAGACCCUGGCAGA
si*Control*	UAAGGCUAUGAAGAGAUAC
	AUGUAUUGGCCUGUAUUAG
	AUGAACGUGAAUUGCUCAA
	UGGUUUACAUGUCGACUAA
**Primer pairs**	**Sequence (5‘ – 3‘)**
Pparg	CACAAGAGCTGACCCAATGGT
	GATCGCACTTTGGTATTCTTGGA
Cebpa	GAGCTGAGTGAGGCTCTCATTCT
	TGGGAGGCAGACGAAAAAAC
Lpl	CCCGTTACCGTCCATCCAT
	GACTCTGTGTCTAACTGCCACTTCA
Runx2	CTTCTGTCTGTGCCTTCTTGGTT
	CAAGTAGCCAGGTTCAACGATCT
Sp7	GGTCACCCCATCTTTGGGA
	GAGCAGAGTGCCAAGAAGGG
Bglap	TTGGCATCTGTGAGGTCAGAGA
	TGAGGACCATCTTTCTGCTCACT
Ahr	TCCCACATCCGCATGATTAA
	TTTGCAAGAAGCCGGAAAA
Rpl13a	TGGTCCCTGCTGCTCTCAA
	CCCCAGGTAAGCAAACTTTCTG
Glis1	TCATCCACATGAGGGTACACTCA
	CAGGTTCTCCAGACGGGAAA
Glis1 *(exogenous)*	CATGTTCGAGGGCTGTAGCA
	TGTGGCTTCTCAGGTGGATCTT
Notch1	CTACCTGCACTGACTATCTC
	GCAGGAACACTTGTAGGA
Notch2	CTGAGTGATGAAGACGAAGA
	CAGCATCAGCTCTCGAATA
Notch3	CCCTCGTATGTACCAAGTAG
	CTGGAGTTGAGGCTTTGA

Supplementary Table S2. List of ENCODE accession numbers of datasets used to validate the affinity threshold for EPIC-DREM analysis.

**GM12878**	**GEO accession number**	**TF / antibody**
1	ENCFF002CGQ	BATF
2	ENCFF002CGU	CEBPB
3	ENCFF002CGV	EBF1
4	ENCFF002CGW	EGR1
5	ENCFF002CGX	ELF1
6	ENCFF002CGY	ETS1
7	ENCFF002CGZ	FOXM1
8	ENCFF002CHA	GABPA
9	ENCFF939TZS	JUNB
10	ENCFF002CHC	MEF2A
11	ENCFF002CHG	NFIC
12	ENCFF002CHJ	PAX5
13	ENCFF002CHH	REST
14	ENCFF002CHT	RXRA
15	ENCFF002CHV	SP1
16	ENCFF002CHQ	SPI1
17	ENCFF002CHW	SRF
18	ENCFF002CHZ	TCF12
19	ENCFF002CIA	TCF3
20	ENCFF144PGS	TCF7
21	ENCFF002CIB	USF1
22	ENCFF002CIC	YY1
23	ENCFF002CID	ZBTB33
24	ENCFF002CIE	ZEB1
25	ENCFF001SUG	H3K27ac (BAM)
26	ENCFF804NCH	H3K27ac (BED)
**K562**	**GEO accession number**	**TF / antibody**

1	ENCSR000BRQ	CEBPB
2	ENCSR000DWE	CTCF
3	ENCSR000BLI	E2F6
4	ENCSR000BNE	EGR1
5	ENCSR000BMD	ELF1
6	ENCSR000BKQ	ETS1
7	ENCSR000BMV	FOSL1
8	ENCSR000BLO	GABPA
9	ENCSR000BKM	GATA2
10	ENCSR000EFV	MAX
11	ENCSR000BNV	MEF2A
12	ENCSR000BMW	REST
13	ENCSR000BKO	SP1
14	ENCSR000BGW	SPI1
15	ENCSR000BLK	SRF
16	ENCSR000BKT	USF1
17	ENCSR000BKU	YY1
18	ENCSR000BKF	ZBTB33
19	ENCFF301TVL	H3K27ac (BAM)
20	ENCFF001SZE	H3K27ac (BED)
**HepG2**	**GEO accession number**	**TF / antibody**
1	ENCFF002CTS	ARID3A
2	ENCSR000BID	BHLHE40
3	ENCFF002CTU	BRCA1
4	ENCFF002CTV	CEBPB
5	ENCSR000DUG	CTCF
6	ENCSR000BMZ	ELF1
7	ENCFF002CUA	ESRRA
8	ENCSR000BHP	FOSL2

9	ENCSR000BMO	FOXA1
10	ENCSR000BNI	FOXA2
11	ENCSR000BJK	GABPA
12	ENCSR000BLF	HNF4A
13	ENCSR000BNJ	HNF4G
14	ENCFF002CUD	HSF1
15	ENCFF002CTY	JUN
16	ENCSR000BGK	JUND
17	ENCFF002CUG	MAFF
18	ENCFF002CUI	MAFK
19	ENCFF002CUJ	MAX
20	ENCSR000BQX	NFIC
21	ENCFF002CUY	NR2C2
22	ENCFF002CUM	NRF1
23	ENCSR000BOT	REST
24	ENCFF002CUT	RFX5
25	ENCSR00BHU	RXRA
26	ENCSR000BJX	SP1
27	ENCSR000BOU	SP2
28	ENCFF002CUV	SREBF1
29	ENCFF001VLB	SREBF2
30	ENCSR000BLV	SRF
31	ENCFF002CUW	TBP
32	ENCSR200BJG	TCF12
33	ENCFF002CUX	TCF7L2
34	ENCSR000BGM	USF1
35	ENCFF002CUZ	USF2
36	ENCSR000BHR	ZBTB33
37	ENCFF001SWK	H3K27ac (BAM)
38	ENCFF805KGN	H3K27ac (BED)

Supplementary Table S3. Differentially expressed genes across the time series of adipocyte and osteoblast differentiation. The Ensembl IDs, gene symbols, sequencing depth-normalized read counts, log_2-fold change and statistical significance of genes with FDR < 0.05 as derived by DEseq2 are listed. Each worksheet corresponds to one time point of differentiation as indicated.

Supplementary Table S4. Benchmarking of the different GRN reconstruction methods with existing literature. The top TFs identified by the indicated method (DREM2.0, EPIC-DREM, DREM-TRAP and random shuffling of the TF-target matrix) are listed per lineage and marked if existing literature supports their predicted role. If yes, at least one reference is provided.

Supplementary Table S5. TFs predicted to control the split points during adipocyte and osteoblast differentiation. List of all TFs predicted to control genes in the indicated time point-specific paths with DREM path significance conditional on split using score < 0.01 and minimum split % of 30. The gene symbols, split scores, Ensembl IDs, log_2-fold changes of the TFs, and the adjusted p-value of the fold change (relative to day 0) are indicated. Each worksheet corresponds to the TFs per path as indicated in Figure 3.

Supplementary Table S6. List of EPIC-DREM predicted PPARG:RXRA target genes. The Ensembl IDs and the affinity scores of the 3405 predicted PPARG:RXRA target genes on day 3 of adipocyte differentiation.

Supplementary Table S7. List of the merged super-enhancers. The coordinates and names of the merged SEs identified across adipocyte and osteoblast differentiation.

Supplementary Table S8. List of the dynamic merged super-enhancers. The dynamic SEs ($log_2FC \geq 1$) and the Ensembl IDs and names of the genes located with +/- 500 kb and showing the highest Pearson correlation with those SEs in the respective lineage are listed.

Supplementary Table S9. Differentially expressed genes following AHR-KD. The Ensembl IDs, gene symbols, sequencing depth-normalized read counts, log_2-fold change and statistical significance of genes with FDR < 0.1 as derived by DEseq2 are listed. Each worksheet corresponds to one knock-down condition.

Supplementary References

1. Wang, Y. *et al.* The 3D Genome Browser: a web-based browser for visualizing 3D genome organization and long-range chromatin interactions. *bioRxiv* (2017).
2. Rao, S.S. *et al.* A 3D map of the human genome at kilobase resolution reveals principles of chromatin looping. *Cell* **159**, 1665-1680 (2014).
3. Dixon, J.R. *et al.* Topological domains in mammalian genomes identified by analysis of chromatin interactions. *Nature* **485**, 376-380 (2012).
4. Bernstein, B.E. *et al.* The NIH Roadmap Epigenomics Mapping Consortium. *Nat Biotechnol* **28**, 1045-1048 (2010).
5. Schmidl, C., Rendeiro, A.F., Sheffield, N.C. & Bock, C. ChIPmentation: fast, robust, low-input ChIP-seq for histones and transcription factors. *Nat Methods* **12**, 963-965 (2015).
6. Chapuy, B. *et al.* Discovery and characterization of super-enhancer-associated dependencies in diffuse large B cell lymphoma. *Cancer cell* **24**, 777-790 (2013).
7. Pacis, A. *et al.* Bacterial infection remodels the DNA methylation landscape of human dendritic cells. *Genome Res* **25**, 1801-1811 (2015).
8. Andersson, R. *et al.* An atlas of active enhancers across human cell types and tissues. *Nature* **507**, 455-461 (2014).
9. Lister, R. *et al.* Human DNA methylomes at base resolution show widespread epigenomic differences. *Nature* **462**, 315-322 (2009).
10. Mei, S. *et al.* Cistrome Data Browser: a data portal for ChIP-Seq and chromatin accessibility data in human and mouse. *Nucleic Acids Res* **45**, D658-d662 (2017).
11. Robinson, J.T. *et al.* Integrative genomics viewer. *Nat Biotech* **29**, 24-26 (2011).

12. Thorvaldsdottir, H., Robinson, J.T. & Mesirov, J.P. Integrative Genomics Viewer (IGV): high-performance genomics data visualization and exploration. *Brief Bioinform* **14**, 178-192 (2013).
